# Anthropomorphic cueing, social evaluation, and threat in stated willingness to collaborate with AI

**DOI:** 10.3389/fpsyg.2026.1896807

**Published:** 2026-09-01

**Authors:** Deyu Yan

**Affiliations:** Advanced Institute for Confucian Studies, Shandong University, Jinan, China

**Keywords:** anthropomorphic cueing, competence, humanlike appearance, realistic threat, stated collaboration intention, uniqueness threat, warmth

## Abstract

AI systems are increasingly presented as team members, but humanlike cues may not uniformly increase willingness to collaborate. We examined how assigned cue packages are related to stated collaboration intention, social evaluations, and threat. Across six two-condition studies (*N* = 1,128), we examined textual cues, imagination instructions, and lower- versus higher-ABOT image-set assignments in imagined collaboration contexts. We distinguished assigned cues from subjective anthropomorphism and measured intention before warmth and competence. Higher-cue conditions increased intention in Studies 1b, 2, 3a, and 3b (*d* = 0.36–0.68), but not in Studies 1a or 4. Bootstrap 95% CIs excluded zero for positive warmth products in five studies, positive competence products in Studies 2 and 3a, and a negative competence product in Study 4. As evaluations followed the outcome, these products are descriptive decompositions rather than causal mediation effects. The exploratory Study 3 comparison could not isolate threat because article, study form, and sample were confounded. The intention interaction was not detectable. In Study 4, the higher-ABOT image set yielded higher subjective anthropomorphism and lower competence, with no detectable overall intention difference. The image-set coefficient became more negative as post-outcome uniqueness threat increased (*b* = −0.396, *p* = 0.008). This interaction remained detectable in an image-fixed-effects sensitivity model conditional on 12 recorded image codes. Assigned cue packages yielded distinct within-study patterns of social evaluation and intention, but did not establish a general benefit of anthropomorphism, a causal threat process, or changes in collaborative behavior.

## 1 Introduction

Artificial intelligence now recommends, evaluates, writes, and participates in tasks once assigned only to human team members. Technical capability does not determine whether people will accept such systems as collaborators. Users can resist systems that seem opaque, socially misplaced, or difficult to hold accountable even when performance is strong (Shariff et al., 2017; Yeomans et al., 2019). Human and AI teaming therefore raises a social judgment problem alongside the engineering problem (O'Neill et al., 2022; Vaccaro et al., 2024). This research asks how cues that invite a human interpretation relate to stated willingness to work with an AI, and where that relation becomes uncertain.

Anthropomorphism refers to the attribution of human properties, motives, intentions, or mental states to a non-human entity (Epley et al., 2007). In AI and human-robot interaction research, however, the same label has often been used for features built into a system and for inferences made by users. A scoping review of 57 studies documented substantial variation in both definition and measurement (Damholdt et al., 2023). Phillips and de Visser (2026) address this problem by treating humanlikeness as a design property and anthropomorphism as a psychological attribution. We follow that distinction. Anthropomorphic cueing refers to language, role framing, behavior, or instructions that invite a human interpretation. Humanlike appearance refers to physical resemblance. Subjective psychological anthropomorphism refers to a user’s attribution of human qualities or mental states. A cue can invite such an inference without ensuring that it occurs.

The Computers Are Social Actors (CASA) framework explains why even sparse cues may elicit a social response. People apply politeness, reciprocity, personality expectations, and social categories to computers without necessarily believing that the machine is human (Nass et al., 1994; Nass and Moon, 2000). Contemporary users may also draw on scripts learned through repeated contact with media technologies (Gambino et al., 2020). Team framing can strengthen affiliation with a computer and openness to its influence (Nass et al., 1996). This framework explains why language and role cues can make an AI socially legible. It does not show that a brief response to a cue is equivalent to subjective anthropomorphism or sustained collaboration.

Mind perception provides a second step in the argument. Observers commonly distinguish experience, which concerns a capacity to feel, from agency, which concerns a capacity to plan and act (Gray et al., 2007; Waytz et al., 2010). Both capacities can be attributed to robots, and interaction style can change those judgments (Cucciniello et al., 2023). Mind perception is related to psychological anthropomorphism, but it is not the same as the warmth and competence judgments studied here. Experience and agency concern whether a target possesses mental capacities. Warmth and competence concern whether its intentions seem benevolent and whether it is able to carry them out. The present studies did not measure experience or agency as intervening variables. Mind perception is used here to explain why social evaluation may become applicable to an AI target.

Research on robot appearance makes these distinctions especially important. Humanlike appearance is multidimensional, with contributions from surface features, body and manipulators, facial features, and mechanical locomotion (Phillips et al., 2018). Physical attributes contribute differently to first impressions of warmth and competence (Reeves et al., 2020). Humanlike appearance can prompt perspective taking even when observers do not grant a robot a rich mind (Zhao and Malle, 2022). Its association with danger to the human–machine boundary can also differ from its association with mind attribution (Müller et al., 2021). Work across 251 robot images found non-linear valleys in emotional response that were associated with mismatches among appearance features (Kim et al., 2022). Appearance should therefore not be treated as a stronger dose of psychological anthropomorphism. It also carries realism, familiarity, function, and category boundary signals.

Once an AI is construed as a social target, the stereotype content model specifies the content of evaluation. Warmth captures perceived intention, while competence captures the capacity to enact that intention (Fiske et al., 2002, 2007). People apply both dimensions to AI systems and prospective AI teammates (Harris-Watson et al., 2023; McKee et al., 2023). Stronger anthropomorphic cueing may therefore change stated collaboration intention alongside these two evaluations. H1 predicted that stronger textual or imagined anthropomorphic cueing would increase stated collaboration intention. H2a predicted higher warmth and competence ratings. H2b predicted that condition differences in warmth and competence would statistically account for part of the condition difference in stated collaboration intention. The last prediction concerns a regression decomposition. It does not establish temporal mediation because the intention measure preceded the two evaluations in every study.

The stereotype content model and intergroup threat theory answer different questions. The first describes how a target is evaluated. The second addresses when an outgroup is understood as harmful to material interests or group identity (Stephan et al., 2016). Realistic threat concerns jobs, safety, resources, and status. Fear of automation and job replacement is one example in an AI setting (McClure, 2018). Symbolic threat concerns values and identity. Threat to human uniqueness is a narrower identity concern that arises when humanlike machines appear to weaken the distinction between humans and machines (Ferrari et al., 2016). It is not interchangeable with every form of symbolic threat.

The two-threat predictions require separate treatment. H3a predicted that the association between anthropomorphic cueing and stated collaboration intention would be weaker in a high realistic threat context. Studies 3a and 3b used independent samples and questionnaire forms within the same recorded date range, so their comparison can only provide exploratory evidence for this prediction. H3b predicted that the association between humanlike appearance and stated collaboration intention would become less positive or more negative as reported uniqueness threat increased. Uniqueness threat was measured after the outcome in Study 4. That analysis therefore describes a correlational boundary pattern rather than causal moderation.

Six studies examined passive text, active imagination, and robot appearance. Their manipulations were not matched for positivity, vividness, number of cues, or participant effort. Only an instruction-level summary survives for Study 2, so the content of its high and low prompts cannot be audited in full. The preserved Study 3 prompts differed in emotion, agency, communication, adaptability, and expected performance as well as human framing. We therefore treat Study 2 as a range-guided imagination contrast with an incomplete prompt record and Studies 3a and 3b as bundled active-imagination contrasts. The studies show how the assigned conditions operated in their particular settings. They do not rank cue types or isolate a pure effect of anthropomorphism.

## 2 Overview and analytic strategy

The original study reports describe Chinese participants recruited through Credamo and a two-condition between-participants design. The cleaned files also carry a paper-questionnaire double-entry label. The surviving records do not show whether that label refers to questionnaire administration, later transcription, or both. We retain the reported recruitment source while disclosing this unresolved provenance field. Participants recalled a current or prior team before evaluating a prospective AI member. Condition assignment was balanced within each retained file. The cleaned files contained 1,128 cases in total. The archived allocation log contained 1,229 identifiers. The 101 identifiers absent from the cleaned files could be counted by study, but the surviving materials did not preserve person-level exclusion reasons. We report the analyzable samples and the discrepancy directly. We do not reconstruct exclusion categories that cannot be verified.

The common outcome was stated collaboration intention, measured with four items adapted from the psychological acceptance dimension of the Human–AI Teams (HATS) newcomer acceptance scale (Harris-Watson et al., 2023). Warmth and competence were each measured with five items from the Perceived AI Warmth and Competence Scale (Hu et al., 2021). Items used seven-point response scales. The intention measure was always completed before warmth and competence. The archive labels the 100-point checks in Studies 1b to 4 as subjective anthropomorphism, but their exact single-item wording was not recovered. We retain that archive label while treating its construct interpretation cautiously. Table 1 summarizes the designs and their inferential limits. Table 2 reports the manipulation checks. Full surviving prompts, item wording, procedure order, scoring rules, and participant flow are provided in the Supplementary material.

**Table 1:** Design and inferential scope of the six studies.

Study	*N*	Assigned factor	Procedure relevant to inference	Primary boundary
1a	278	Text cueing in an evaluation scenario	Cue, check, intention, warmth, competence	Evaluations followed the outcome
1b	180	Text cueing in general collaboration	Cue, check, intention, warmth, competence	Evaluations followed the outcome
2	136	Range-guided imagination	Baseline, range instruction, check, intention, evaluations	Full condition wording was not preserved
3a	178	Bundled imagination with lower threat article	Anthropomorphism Questionnaire, package, article, threat, intention, evaluations	Threat fixed by study, form, and sample
3b	178	Bundled imagination with higher threat article	Anthropomorphism Questionnaire, package, article, threat, intention, evaluations	Threat fixed by study, form, and sample
4	178	Lower- or higher-ABOT image set	Image, check, intention, evaluations, uniqueness threat	Moderator followed outcome. Image nested in condition

**Table 2:** Manipulation check results.

Study	Scale	Low M (SD)	High M (SD)	*t*(df)	*p*	*d*
1a	1–7	4.20 (1.10)	4.59 (1.12)	2.92 (276)	0.004	0.35
1b	1–100	48.52 (23.13)	58.07 (23.68)	2.74 (178)	0.007	0.41
2	1–100	31.96 (17.45)	68.56 (18.13)	11.99 (134)	<0.001	2.06
3a	1–100	34.17 (18.26)	73.49 (16.08)	15.25 (176)	<0.001	2.29
3b	1–100	27.67 (17.61)	71.97 (15.04)	18.05 (176)	<0.001	2.71
4	1–100	32.93 (15.55)	63.87 (17.60)	12.43 (176)	<0.001	1.86

The checks used different scales and cue packages. Their effect sizes do not provide a controlled comparison of cue type.

Condition was coded 0 for the low condition and 1 for the high condition. Two-sided, pooled-variance independent-samples t-tests compared the manipulation check and stated collaboration intention, warmth, and competence. Cohen’s d uses the high minus low direction. Manipulation checks were treated as design checks. The Benjamini–Hochberg procedure controlled the false discovery rate across the 18 focal condition tests of intention, warmth, and competence (Benjamini and Hochberg, 1995). Raw *p*-values and adjusted q-values are reported together.

Parallel indirect statistical associations were estimated by ordinary least squares with 5,000 case-resampling bootstrap draws and percentile confidence intervals. The primary decomposition was unadjusted in every study. This choice avoided selecting covariates from sample correlations and matched the unadjusted randomized condition contrasts. Design-relevant or previously used adjustments for the NAO baseline, anthropomorphism tendency, and gender indicators were retained as sensitivity analyses. Those models led to the same substantive decisions. The bootstrap products are descriptive statistical decompositions because the proposed intermediates followed the outcome in the questionnaire.

Studies 3a and 3b were also combined in an exploratory model containing cue condition, a study indicator, and their interaction. Study, independent sample, questionnaire form, and threat context were perfectly aligned. No coefficient from that model identifies a causal threat effect. Study 4 used an ordinary least squares model with centered uniqueness threat and its interaction with the assigned higher- versus lower-ABOT image set. The primary model was unadjusted. Gender indicators were added in a sensitivity model. A further model absorbed image fixed effects. As every image belonged to only one image-set condition, the fixed-effects model cannot identify the image-set main coefficient, and its uncertainty is conditional on the 12 recorded image codes.

## 3 Study 1. Passive textual cueing

Study 1 examined two restrained textual manipulations. Study 1a placed the AI in an employee evaluation task. Study 1b used a broader collaboration scenario. Both manipulations changed the target’s name, role description, and linguistic framing while holding the assigned task constant.

### 3.1 Study 1a

#### 3.1.1 Method

The cleaned Study 1a file contained 278 participants, with 139 in each condition. The sample included 183 women, 90 men, and 5 participants who selected another gender category. Mean age was 31.82 years (SD = 8.41). The allocation log contained 302 identifiers, 24 more than the cleaned file. The archived records did not preserve the reason attached to each absent identifier. The original sample size plan targeted a medium between-condition effect of *d* = 0.50 with 90% power and alpha of 0.05, which required at least 172 participants (Faul et al., 2009). This calculation did not establish power for indirect associations.

Participants first described a reference team. They then read that an AI member would help with employee performance evaluation. In the stronger cue condition, the AI had a human name, used first-person language, and was described as a human resource assistant. In the weaker cue condition, it had an instrumental label and was described as an algorithm. Participants completed a seven-point humanlikeness check, stated collaboration intention, warmth, competence, an attention check, and demographics in that order. Reliability was 0.812 for intention, 0.880 for warmth, and 0.822 for competence.

#### 3.1.2 Results

The cue increased the humanlikeness rating. The high condition produced M = 4.59 and SD = 1.12 compared with M = 4.20 and SD = 1.10 in the low condition, *t*(276) = 2.92, *p* = 0.004, *d* = 0.35. Warmth was also higher in the high condition, M = 4.43 and SD = 1.26, than in the low condition, M = 4.04 and SD = 1.34, *t*(276) = 2.47, *p* = 0.014, *q* = 0.028, *d* = 0.30. Competence did not differ, *t*(276) = −0.41, *p* = 0.681, *q* = 0.681. Stated collaboration intention also did not differ. The high condition produced M = 4.09 and SD = 1.21 compared with M = 4.16 and SD = 1.31 in the low condition, *t*(276) = −0.42, *p* = 0.677, *q* = 0.681, *d* = −0.05.

The primary bootstrap decomposition showed a positive warmth product, estimate = 0.115, 95% CI [0.022, 0.228]. The competence product included zero, estimate = −0.018, 95% CI [−0.117, 0.064]. The remaining condition coefficient also included zero, *b* = −0.160, 95% CI [−0.429, 0.107]. Study 1a therefore showed a change in warmth without a detectable condition difference in stated intention.

### 3.2 Study 1b

#### 3.2.1 Method

The cleaned Study 1b file contained 180 participants, with 90 in each condition. The sample included 105 women, 71 men, and 4 participants in another gender category. Mean age was 31.22 years (SD = 8.00). The allocation log listed 194 identifiers, 14 more than the cleaned file. Study 1b used the same sample size target as Study 1a.

Participants read about either an intelligent collaboration partner named Archie or an instrumental collaboration tool labeled HAP. They then completed a 100-point subjective anthropomorphism check. The remaining measures and their order matched Study 1a. Reliability was 0.804 for intention, 0.878 for warmth, and 0.847 for competence. The full condition texts are reproduced in the Supplementary material.

#### 3.2.2 Results

Subjective anthropomorphism was higher in the stronger cue condition, M = 58.07 and SD = 23.68, than in the weaker cue condition, M = 48.52 and SD = 23.13, *t*(178) = 2.74, *p* = 0.007, *d* = 0.41. Warmth also increased. The stronger cue condition produced M = 4.64 and SD = 1.32, compared with M = 4.01 and SD = 1.35 in the weaker cue condition, *t*(178) = 3.17, *p* = 0.002, *q* = 0.005, *d* = 0.47. Competence did not differ, *t*(178) = 0.66, *p* = 0.510, *q* = 0.574. Unlike Study 1a, stated collaboration intention was higher in the stronger cue condition, M = 4.37 and SD = 1.23, than in the weaker cue condition, M = 3.92 and SD = 1.26, *t*(178) = 2.41, *p* = 0.017, *q* = 0.028, *d* = 0.36.

The unadjusted warmth product was positive, estimate = 0.157, 95% CI [0.045, 0.318]. The competence product included zero, estimate = 0.030, 95% CI [−0.073, 0.122]. The remaining condition coefficient also included zero, *b* = 0.260, 95% CI [−0.070, 0.588]. Adding gender indicators changed the warmth estimate to 0.142, 95% CI [0.033, 0.299] but did not change the inference. Taken together, the two text studies consistently changed warmth. Only Study 1b showed a detectable condition difference in stated collaboration intention.

## 4 Study 2. Range-guided imagination with an incomplete prompt record

### 4.1 Method

The cleaned Study 2 file contained 136 participants, with 68 in each condition. The sample included 92 women, 41 men, and 3 participants in another gender category. Mean age was 31.17 years (SD = 7.86). The allocation log contained 148 identifiers, 12 more than the cleaned file. The original sample plan targeted *d* = 0.50 with 80% power and required at least 128 participants. It was not a power analysis for an indirect association.

Participants first rated a NAO robot image on a 100-point humanlikeness scale. The surviving supplement states that participants were then instructed to imagine a robot partner within either a lower range from 1 to 33 or a higher range from 67 to 100. It preserves no verbatim Study 2 condition text beyond that instruction-level summary. The available check establishes that the assigned imagination conditions produced different subjective anthropomorphism ratings. It does not reveal the full wording of the prompts or identify which unrecorded features contributed to the outcome differences. We do not import the more detailed Study 3 prompt content into Study 2.

After the imagination task, participants completed the 100-point subjective anthropomorphism check, stated collaboration intention, warmth, competence, an attention check, and demographics. Reliability was 0.815 for intention, 0.888 for warmth, and 0.838 for competence. The surviving instruction summary and its evidence limits are reported in the Supplementary material.

### 4.2 Results

Baseline NAO ratings did not differ. The high condition produced M = 47.84 and SD = 23.03 compared with M = 45.03 and SD = 23.99 in the low condition, *t*(134) = 0.70, *p* = 0.487, *d* = 0.12. After the imagination task, subjective anthropomorphism was substantially higher in the high condition, M = 68.56 and SD = 18.13, than in the low condition, M = 31.96 and SD = 17.45, *t*(134) = 11.99, *p* < 0.001, *d* = 2.06.

The higher-range condition produced higher stated collaboration intention, M = 4.33 and SD = 1.24, than the lower-range condition, M = 3.83 and SD = 1.22, *t*(134) = 2.36, *p* = 0.020, *q* = 0.030, *d* = 0.40. Warmth was higher in the higher-range condition, M = 4.88 and SD = 1.32, than in the lower-range condition, M = 3.96 and SD = 1.27, *t*(134) = 4.14, *p* < 0.001, *q* < 0.001, *d* = 0.71. Competence followed the same pattern. The higher-range condition produced M = 4.40 and SD = 1.16, compared with M = 3.89 and SD = 1.28 in the lower-range condition, *t*(134) = 2.42, *p* = 0.017, *q* = 0.028, *d* = 0.42.

Both bootstrap products excluded zero. The warmth estimate was 0.199, 95% CI [0.045, 0.382]. The competence estimate was 0.153, 95% CI [0.022, 0.329]. The remaining condition coefficient was 0.145, 95% CI [−0.258, 0.553]. Including the NAO baseline produced almost identical estimates. These results describe the statistical composition of the recorded Study 2 contrast. They cannot attribute the pattern to a particular prompt component or establish that the later evaluations carried an earlier effect on intention.

## 5 Study 3. Two preregistered imagination studies in different threat contexts

Studies 3a and 3b were preregistered together under AsPredicted registration no. 243,886.^1^ Each study randomized the bundled imagination condition within its own sample. The archived registration lists four stated collaboration intention items whose English phrasing is not identical to the wording preserved in the archived questionnaire documentation. The present analyses use the four scored item variables in the cleaned files, and the Supplementary material documents this wording discrepancy. Study 3a used a lower realistic threat article, and Study 3b used a higher realistic threat article. Both cleaned records cover the same date range, but the threat articles belonged to different study forms and independent samples. The two studies can test the cue package within each sample. Their comparison cannot identify the effect of realistic threat.

### 5.1 Study 3a

#### 5.1.1 Method

The cleaned Study 3a file contained 178 participants, with 89 in each cue condition. The sample included 100 women, 77 men, and 1 participant in another gender category. Mean age was 29.99 years (SD = 7.73). The allocation log contained 194 identifiers, 16 more than the cleaned file. The preregistered target was approximately 172 participants, based on a medium independent-groups effect with 90% power.

Participants completed five electronic product items from the Anthropomorphism Questionnaire (Neave et al., 2015). They then received the high or low imagination prompt and supplied three descriptions covering appearance and movement, personality and emotion, and behavior. The stored open-response fields contain coded placeholders rather than verbatim responses, so the qualitative descriptions cannot be reanalyzed. Participants next completed the subjective anthropomorphism check, read the lower threat article, and completed realistic threat and ingroupness measures, including an adapted Inclusion of Other in the Self item (Aron et al., 1992). Stated collaboration intention, warmth, competence, attention checks, and demographics followed. Reliability was 0.809 for anthropomorphism tendency, 0.864 for realistic threat, 0.729 for ingroupness, 0.850 for intention, 0.878 for warmth, and 0.852 for competence.

#### 5.1.2 Results

The high package increased subjective anthropomorphism, M = 73.49 and SD = 16.08, relative to the low package, M = 34.17 and SD = 18.26, *t*(176) = 15.25, *p* < 0.001, *d* = 2.29. Stated collaboration intention was higher in the high condition, M = 4.57 and SD = 1.33, than in the low condition, M = 3.69 and SD = 1.28, *t*(176) = 4.52, *p* < 0.001, *q* < 0.001, *d* = 0.68. Warmth increased by 1.11 scale points, *t*(176) = 5.99, *p* < 0.001, *q* < 0.001, *d* = 0.90. Competence increased by 0.76 points, *t*(176) = 4.06, *p* < 0.001, *q* < 0.001, *d* = 0.61.

The warmth product was 0.467, 95% CI [0.267, 0.707]. The competence product was 0.216, 95% CI [0.089, 0.373]. The remaining condition coefficient included zero, *b* = 0.199, 95% CI [−0.152, 0.535]. Adjustment for anthropomorphism tendency produced the same decisions.

### 5.2 Study 3b

#### 5.2.1 Method

The cleaned Study 3b file also contained 178 participants, with 89 in each cue condition. The sample included 92 women, 82 men, and 4 participants in another gender category. Mean age was 31.67 years (SD = 8.09). The allocation log contained 195 identifiers, 17 more than the cleaned file. Procedures and measures matched Study 3a except for the higher threat article. Reliability was 0.785 for anthropomorphism tendency, 0.868 for realistic threat, 0.652 for ingroupness, 0.828 for intention, 0.869 for warmth, and 0.815 for competence. The lower ingroupness reliability narrows the conclusions that can be drawn from that measure.

#### 5.2.2 Results

Subjective anthropomorphism was higher in the high package, M = 71.97 and SD = 15.04, than in the low package, M = 27.67 and SD = 17.61, *t*(176) = 18.05, *p* < 0.001, *d* = 2.71. Stated collaboration intention was also higher. The high package produced M = 4.33 and SD = 1.28, compared with M = 3.82 and SD = 1.28 in the low package, *t*(176) = 2.62, *p* = 0.010, *q* = 0.022, *d* = 0.39. Warmth increased from M = 3.78 and SD = 1.32 in the low package to M = 4.70 and SD = 1.20 in the high package, *t*(176) = 4.88, *p* < 0.001, *q* < 0.001, *d* = 0.73. Competence did not differ. The high package produced M = 4.27 and SD = 1.15, compared with M = 4.12 and SD = 1.22 in the low package, *t*(176) = 0.81, *p* = 0.421, *q* = 0.505, *d* = 0.12.

The warmth product was positive, estimate = 0.158, 95% CI [0.023, 0.317]. The competence product included zero, estimate = 0.054, 95% CI [−0.077, 0.192]. The remaining condition coefficient also included zero, *b* = 0.291, 95% CI [−0.069, 0.649]. Adjustment for anthropomorphism tendency did not alter these decisions. Study 3b therefore did not reproduce the competence difference observed in Study 3a.

### 5.3 Exploratory cross-study comparison

Reported realistic threat was higher in Study 3b, M = 4.81 and SD = 1.31, than in Study 3a, M = 4.05 and SD = 1.35, *t*(354) = 5.32, *p* < 0.001, *d* = 0.56. This contrast does not constitute a randomized manipulation check. Study membership, independent sample, questionnaire form, and article condition were perfectly aligned.

The cue condition by study interaction was not different from zero for warmth, *b* = −0.191, SE = 0.264, *t*(352) = −0.72, *p* = 0.471, 95% CI [−0.711, 0.329]. It was also not different from zero for stated collaboration intention, *b* = −0.379, SE = 0.274, *t*(352) = −1.38, *p* = 0.167, 95% CI [−0.918, 0.159]. The competence interaction was negative, *b* = −0.616, SE = 0.259, *t*(352) = −2.38, *p* = 0.018, 95% CI [−1.124, −0.107]. The competence condition difference was smaller in Study 3b. The preregistered prediction that the high cue would not increase intention in Study 3b was not supported, and the exploratory between-study difference in the intention contrast was not detectable. Any cross-study difference could arise from the threat article, the independent sample, the questionnaire form, or another study feature. H3a therefore remains untested as a causal claim.

## 6 Study 4. Assigned ABOT image sets and post-outcome uniqueness threat

### 6.1 Method

The cleaned Study 4 file contained 178 participants, with 89 assigned to each ABOT image-set condition. The sample included 100 women, 71 men, and 7 participants in another gender category. Mean age was 32.33 years (SD = 8.72). The allocation log contained 196 identifiers, 18 more than the cleaned file. The original sample plan targeted *d* = 0.50 with 90% power. It did not target the interaction or stimulus-level uncertainty.

Participants viewed one robot image associated in the cleaned file with a lower or higher Anthropomorphic roBOT (ABOT) human-likeness score (Phillips et al., 2018). Six codes occurred in each assigned image set. Retained counts ranged from 12 to 17 per code. The within-condition mechanism used to assign participants to image codes was not preserved. Only retained IDs and counts can be reported. A surviving reference table maps those codes to robot names and ABOT scores. The original presentation record and image redistribution permissions were not recovered. We therefore report the codes, scores, and allocations as archived metadata rather than claim that the available reference files are authenticated copies of the displays. The higher- and lower-ABOT image sets also differed in unmeasured realism, familiarity, attractiveness, visual complexity, likely function, eeriness, and perceived agency.

After image exposure, participants completed a 100-point subjective anthropomorphism check, stated collaboration intention, warmth, competence, and a five-item uniqueness threat scale adapted from perceived robot threat research (Yogeeswaran et al., 2016). Attention and demographic items followed. Uniqueness threat was therefore recorded after the outcome and the two evaluations. Reliability was 0.762 for intention, 0.848 for warmth, 0.816 for competence, and 0.809 for uniqueness threat.

### 6.2 Results

The assigned higher-ABOT image set produced higher subjective anthropomorphism, M = 63.87 and SD = 17.60, than the lower-ABOT set, M = 32.93 and SD = 15.55, *t*(176) = 12.43, *p* < 0.001, *d* = 1.86. Stated collaboration intention did not differ. The higher-ABOT set produced M = 3.90 and SD = 1.23, compared with M = 4.15 and SD = 1.11 in the lower-ABOT set, *t*(176) = −1.42, *p* = 0.157, *q* = 0.218, *d* = −0.21. Warmth did not differ, *t*(176) = 0.87, *p* = 0.385, *q* = 0.495. Competence was lower in the higher-ABOT set, M = 3.70 and SD = 1.21, than in the lower-ABOT set, M = 4.16 and SD = 1.11, *t*(176) = −2.65, *p* = 0.009, *q* = 0.022, *d* = −0.40.

The unadjusted warmth product included zero, estimate = 0.030, 95% CI [−0.043, 0.111]. The competence product was negative, estimate = −0.115, 95% CI [−0.245, −0.018]. The remaining condition coefficient included zero, *b* = −0.165, 95% CI [−0.506, 0.172]. Gender adjustment produced the same decisions and a competence product of −0.101, 95% CI [−0.225, −0.006]. The total condition contrast was not detectable, and the 95% confidence interval for the remaining condition coefficient included zero.

The primary unadjusted boundary model yielded a negative interaction between assigned image set and uniqueness threat, *b* = −0.396, SE = 0.147, *t*(174) = −2.70, *p* = 0.008, 95% CI [−0.686, −0.107], R^2^ = 0.096. The conditional higher-minus-lower image-set coefficient was 0.195 at one standard deviation below the uniqueness mean, *p* = 0.417. It was −0.264 at the mean, *p* = 0.120, and −0.724 at one standard deviation above the mean, *p* = 0.003. The Johnson–Neyman procedure identified 4.114 as the only boundary within both the observed and theoretical ranges (Johnson and Neyman, 1936). Above that value, the conditional coefficient was negative and its 95% interval excluded zero. A second mathematical root at 0.876 was outside the observed range.

The interaction remained after two gender indicators were added, *b* = −0.392, SE = 0.150, *p* = 0.010, and the within-range boundary was 4.172. It also remained in an unadjusted image fixed-effects model, *b* = −0.402, SE = 0.151, *t*(164) = −2.66, *p* = 0.009, 95% CI [−0.700, −0.103]. The image-set main coefficient cannot be estimated in the fixed-effects model because each image is nested in one condition. The sensitivity result applies to the 12 recorded codes. It does not represent uncertainty across the population of robot images.

Taken together, the results of Study 4 support only a narrow conclusion. Assignment to the higher-ABOT image set changed subjective anthropomorphism and coincided with lower competence ratings. The interaction with uniqueness threat describes a post-outcome association. It cannot show that uniqueness threat caused the image-set response. Nor can the design attribute the image-set contrast specifically to human likeness rather than eeriness, realism, familiarity, or perceived function.

### 6.3 Cross-study summary

**Figure 1:**
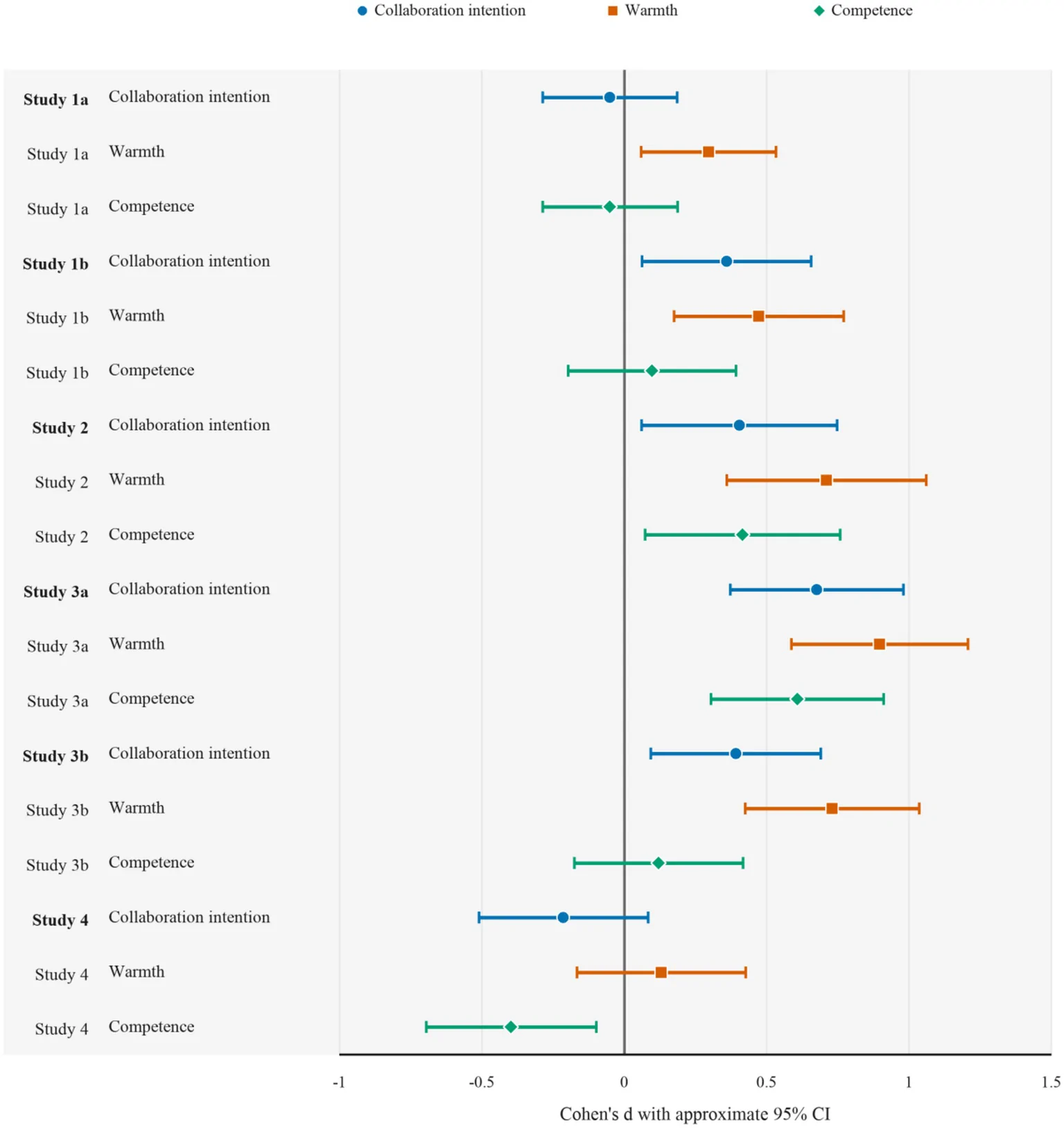
Assigned-condition differences across six studies. Points are Cohen’s *d* estimates with approximate 95% confidence intervals.

**Figure 2:**
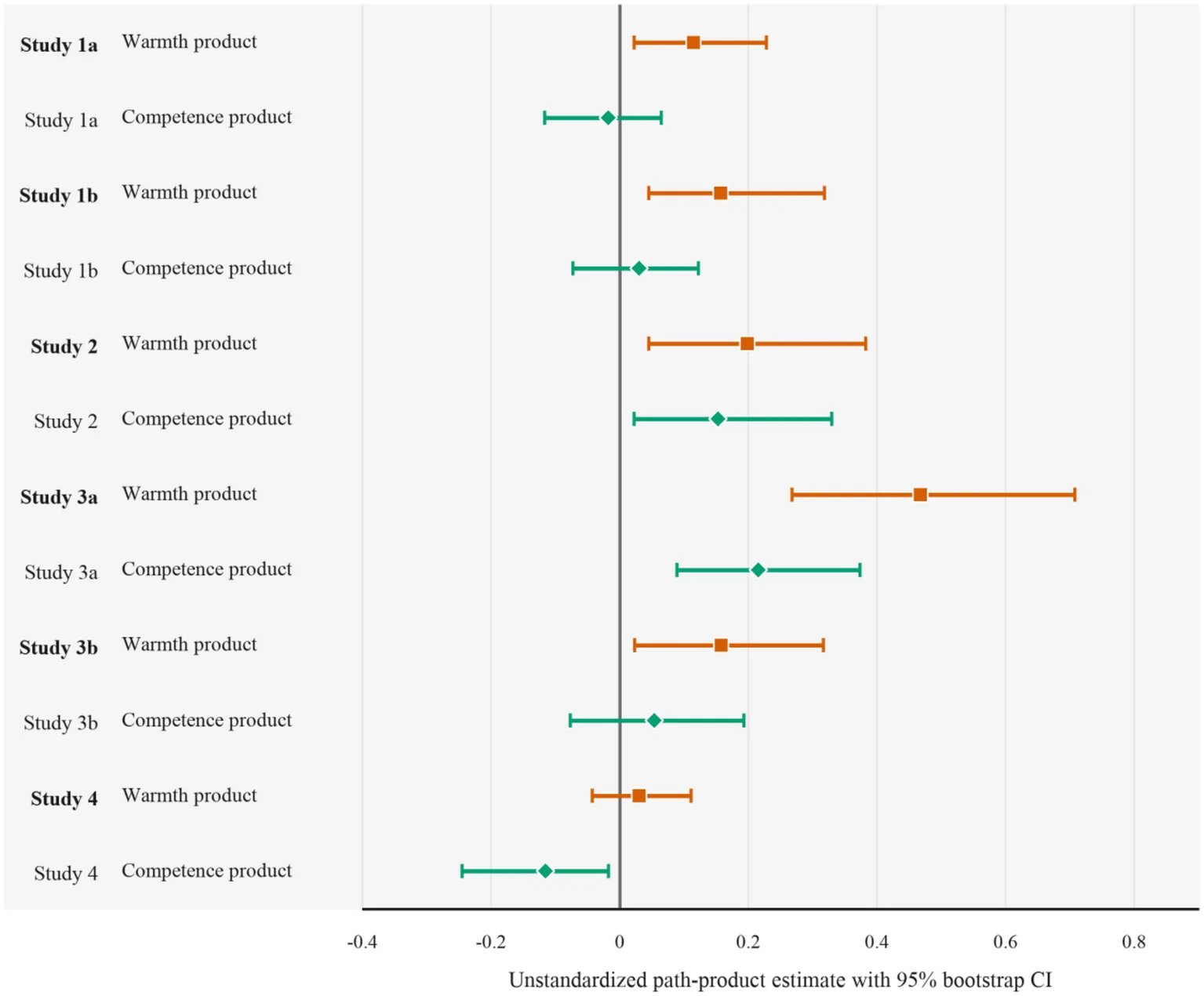
Warmth and competence path-product estimates. Error bars are 95% bootstrap confidence intervals from the primary unadjusted decompositions. The products do not imply causal mediation.

Table 3 reports the primary unadjusted indirect statistical associations. Figure 1 compares the standardized condition differences in stated collaboration intention, warmth, and competence. Figure 2 displays the warmth and competence products with their bootstrap intervals.

**Table 3:** Primary unadjusted parallel indirect statistical associations.

Study	Warmth product [95% CI]	Competence product [95% CI]	Remaining condition *b* [95% CI]
1a	0.115 [0.022, 0.228]	−0.018 [−0.117, 0.064]	−0.160 [−0.429, 0.107]
1b	0.157 [0.045, 0.318]	0.030 [−0.073, 0.122]	0.260 [−0.070, 0.588]
2	0.199 [0.045, 0.382]	0.153 [0.022, 0.329]	0.145 [−0.258, 0.553]
3a	0.467 [0.267, 0.707]	0.216 [0.089, 0.373]	0.199 [−0.152, 0.535]
3b	0.158 [0.023, 0.317]	0.054 [−0.077, 0.192]	0.291 [−0.069, 0.649]
4	0.030 [−0.043, 0.111]	−0.115 [−0.245, −0.018]	−0.165 [−0.506, 0.172]

Intervals are percentile intervals from 5,000 case-resampling bootstrap draws. Stated collaboration intention was measured before warmth and competence. The estimates do not establish temporal or causal mediation.

**Figure 3:**
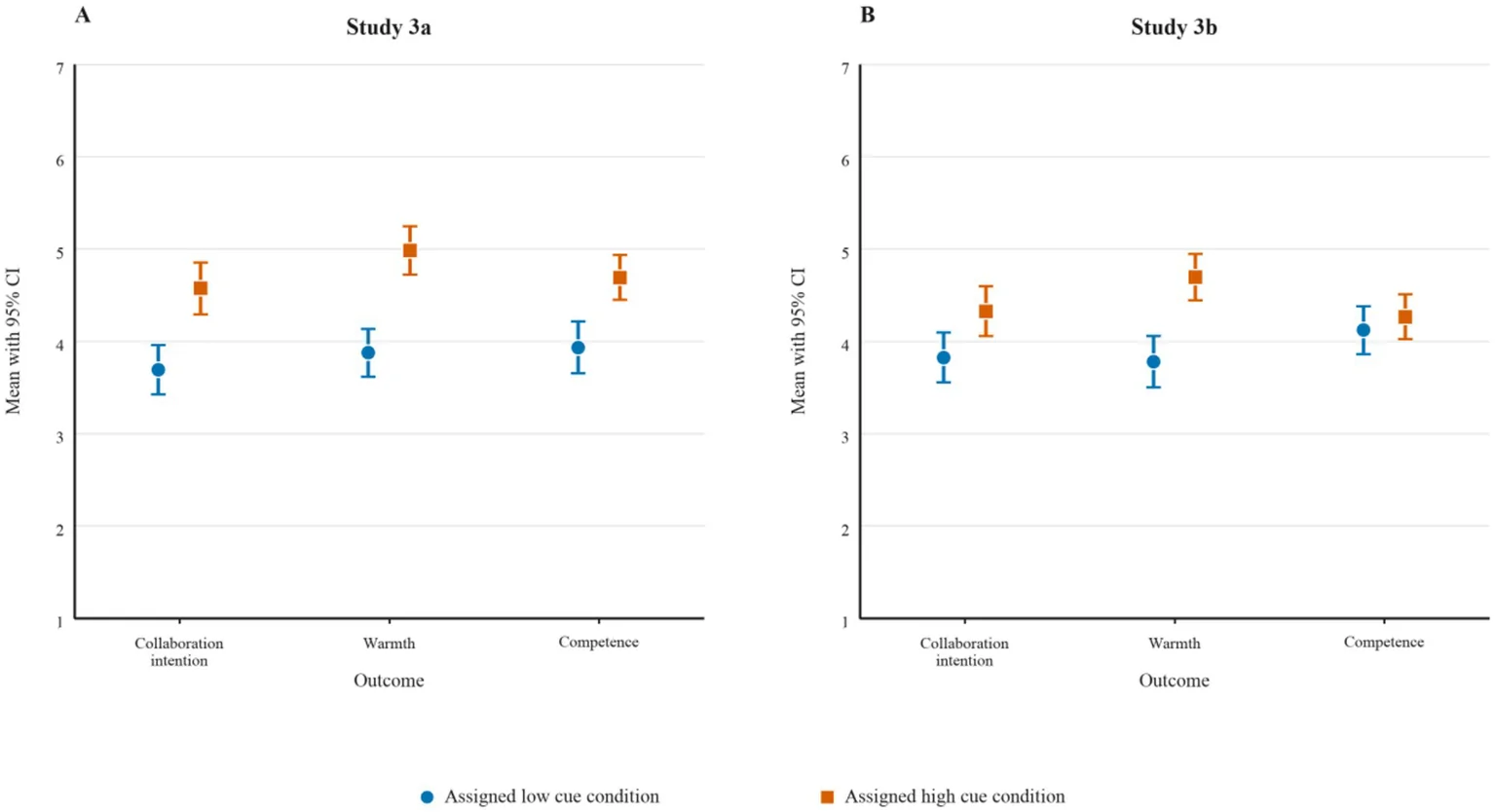
Condition means in **(A)** Study 3a and **(B)** Study 3b. Error bars are 95% confidence intervals.

**Figure 4:**
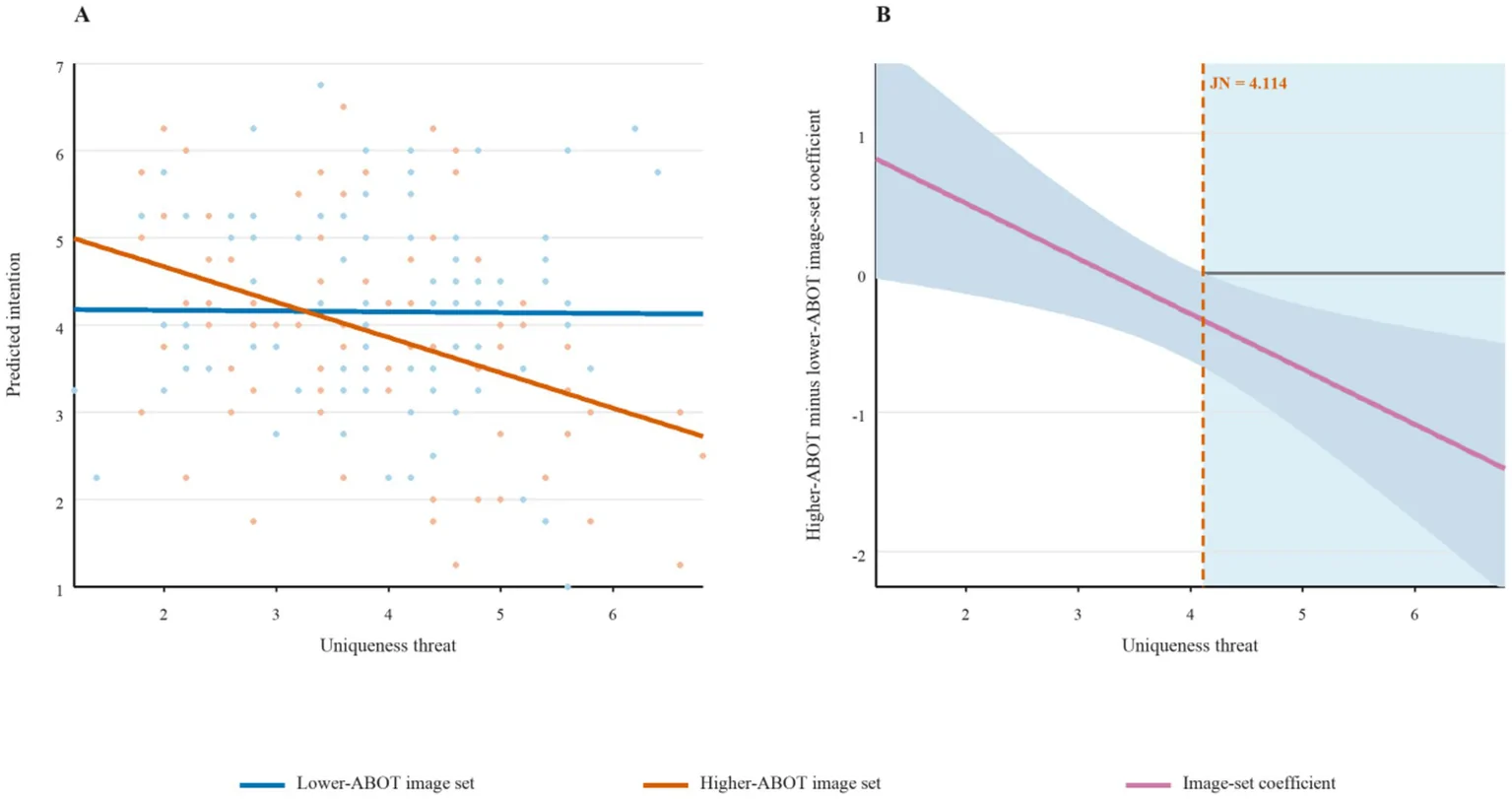
Study 4 image-set moderation. **(A)** Shows predicted intention. **(B)** Shows the conditional image-set coefficient, 95% confidence band, and Johnson–Neyman threshold.

Table 4 consolidates the exploratory boundary analyses and the limits on their interpretation. Figure 3 displays the condition means in the two independent Study 3 samples. Figure 4 shows the Study 4 image-set association across the observed range of uniqueness threat.

**Table 4:** Exploratory boundary analyses.

Analysis	Term	*b*	SE	df	*p*	Interpretive limit
Study 3 cross-study	Warmth cue × study	−0.191	0.264	352	0.471	Study, sample, form, and threat confounded
Study 3 cross-study	Competence cue × study	−0.616	0.259	352	0.018	Study, sample, form, and threat confounded
Study 3 cross-study	Intention cue × study	−0.379	0.274	352	0.167	Study, sample, form, and threat confounded
Study 4 primary	Image set × uniqueness	−0.396	0.147	174	0.008	Uniqueness followed the outcome
Study 4 gender sensitivity	Image set × uniqueness	−0.392	0.150	172	0.010	Two gender indicators included
Study 4 image fixed effects	Image set × uniqueness	−0.402	0.151	164	0.009	Conditional on 12 image codes

## 7 General discussion

The six studies do not yield a simple verdict that humanizing AI helps or harms. They show several patterns tied to different cueing procedures. The text manipulations changed warmth in both studies, while only Study 1b showed a condition difference in stated intention. The range-guided Study 2 condition and the bundled Study 3 conditions changed warmth and intention. Competence changed in Studies 2 and 3a but not Study 3b. Assignment to the higher-ABOT image set changed subjective anthropomorphism, coincided with lower competence, and showed no overall intention difference in Study 4. A post-outcome uniqueness measure marked where the image-set coefficient became negative.

### 7.1 Cues, inferences, and social evaluation

The distinction between a cue and an inference clarifies this variation. CASA explains why a name, role, or image can invite social responding. Mind perception explains why users may attribute feeling or agency. Neither framework implies that every humanlike feature leads to the same judgment. The present results bear this out. Assignment to the higher-ABOT image set changed subjective anthropomorphism but did not reproduce the warmth pattern of the imagination studies. Humanlike design, psychological anthropomorphism, warmth, competence, and stated intention should remain separate constructs.

The imagination results call for two different forms of caution. The Study 2 record preserves only the target human-likeness ranges and not the full condition wording, so the components of that contrast cannot be reconstructed. The Study 3 prompts are more complete. Their high package presented a more emotional, communicative, adaptive, and capable partner, while the low package was mechanical and limited. Higher warmth under that contrast is unsurprising, and the competence pattern may partly reflect expected performance built into the instructions. The data cannot determine how much each component contributed. A stronger manipulation check does not solve either identification problem. Future experiments should preserve the full prompts and vary human framing, emotional responsiveness, agency, and expected performance independently.

### 7.2 Warmth and competence as post-outcome correlates

Across the six studies, warmth was the more consistent evaluation, although its relation to intention was neither uniform nor causal. Its bootstrap product excluded zero in five studies, including both text studies and all three imagination studies. Competence products were positive in Studies 2 and 3a, absent in Studies 1a, 1b, and 3b, and negative in Study 4. This uneven pattern argues against treating warmth and competence as a fixed pathway that follows every anthropomorphic cue. It is more consistent with the stereotype content model’s premise that intention and capacity judgments respond to different information (Fiske et al., 2002, 2007).

The pattern cannot be read causally because the measurement order runs in the opposite direction from the proposed mediation sequence. Participants stated their collaboration intention before rating warmth and competence. The products may reflect shared evaluation, reverse ordering, or a response consistency process. They are useful because they show how the condition difference decomposes statistically. They do not establish that an earlier warmth or competence change carried a later behavioral effect. A stronger test would measure or manipulate social evaluation before the outcome, separate the measurements in time, and include behavioral choices.

### 7.3 Threat claims after the design audit

The evidence for threat is limited in two distinct ways. Studies 3a and 3b used independent samples and different questionnaire forms within the same recorded date range. The competence condition difference varied across them, but warmth and stated intention did not show a detectable interaction. No result can be assigned specifically to realistic threat because article condition, study, form, and sample were aligned. H3a needs a single randomized 2 by 2 experiment in which cueing and threat are independently assigned.

Study 4 presents a different inferential limit because uniqueness threat was measured after the image and after stated intention. Participants’ earlier reactions could have influenced the threat rating. The negative interaction is therefore compatible with several processes. Concern about human uniqueness may shape image evaluation. An unsettling image may heighten reported uniqueness concern. A negative intention judgment may also make later threat ratings more available. Only a prior measure or an independent manipulation can distinguish these accounts.

### 7.4 ABOT image sets and stimulus dependence

Appearance involves more than a human-likeness score. Realism, familiarity, attractiveness, surface finish, visual complexity, likely use, and perceived agency can alter responses to a robot (Mori, 1970; Reeves et al., 2020; Zhao and Malle, 2022; Kim et al., 2022). The image fixed-effect sensitivity analysis shows that the interaction is not explained by a simple mean difference among the 12 recorded codes. It does not generalize the result to other robots. The condition has only six image codes at each level, and image identity is nested within condition. A future stimulus sampling design should use many images, cross key visual attributes, and model image as a sampled factor.

### 7.5 Implications

The findings support questions for design rather than prescriptions. Textual identity cues can change warmth, but their association with stated intention was not identical across two similar studies. The Study 2 imagination condition produced broader differences, yet its full prompt record is unavailable. The Study 3 packages bundled favorable social and performance information. Assignment to the higher-ABOT image set did not produce a detectable increase in stated intention. Designers should therefore test specific cues in the task where they will be used. They should measure actual reliance, information sharing, correction, and joint performance rather than assume that a favorable rating will transfer to teamwork.

Consequential settings demand an even higher standard because these studies did not involve health care, personnel decisions, finance, or safety-critical work. They cannot justify strong personality cues in those settings. Accountability, calibrated competence, understandable roles, and clear responsibility should be established before social styling is evaluated. Whether warmth supports or distracts from those requirements remains an empirical question.

### 7.6 Limitations and future research

Several limits follow directly from the study records. First, all outcomes were stated intentions in imagined or image-based settings. Participants did not cooperate with a working AI. The evidence does not establish changes in advice taking, disclosure, reliance, coordination, or performance. Behavioral replication is essential.

Second, the full Study 2 prompt record was not preserved, so the components of its imagination contrast cannot be verified. The Study 3 active-imagination packages confounded human framing with positivity, agency, emotion, communication, adaptability, and expected performance. The available manipulation checks do not isolate these components. Factorial cue tests and matched pretests are needed. The stored Study 3 description fields also contain coded placeholders rather than verbatim responses, which prevents a qualitative check of what participants actually imagined.

Third, the temporal ordering does not support causal mediation. Warmth and competence followed stated intention. Future work should place the proposed intermediates before the outcome, use temporal separation, manipulate the evaluations where possible, and compare alternative orders. The present bootstrap intervals are not a substitute for that design.

Fourth, the threat tests are limited in different ways. Study 3 confounds threat with study, questionnaire form, and independent sample. Study 4 measures uniqueness threat after the outcome. A credible test requires independent randomization of realistic threat and a prior or manipulated uniqueness construct. It should also distinguish broad symbolic threat from the narrower concern about human distinctiveness.

Fifth, the Study 4 record is incomplete. The cleaned data contain 12 image codes, scores, and allocations, but the available records do not authenticate the image files against the original presentation record or establish redistribution rights. The images also were not matched on competing visual attributes. The fixed-effect sensitivity is conditional on a small set of codes and does not address stimulus population uncertainty.

Sixth, person-level exclusion reasons could not be recovered for the 101 allocation identifiers absent from the cleaned files. The retained files pass internal scoring and sample size checks, but the flow from all allocated cases to the analysis sample cannot be reconstructed by reason. Future data management should retain an immutable exclusion log and the unfiltered source export.

Seventh, the samples came from one Chinese recruitment source. Gender categories were uneven, and the studies were not powered to examine gender interactions. The original power plans targeted medium condition differences. They did not demonstrate adequate sensitivity for indirect products, cross-study interactions, or stimulus-level effects. The false discovery rate adjustment reduces one multiplicity concern, but replication with broader samples remains necessary.

## 8 Conclusion

Anthropomorphic cues did not operate as a single treatment across these six studies. Textual cues were most consistently associated with warmth. The imagination conditions were associated with wider differences in social evaluation and stated collaboration intention, but the Study 2 prompt record is incomplete, and the Study 3 components cannot be separated. The assigned higher-ABOT image set produced a distinct pattern that included lower competence and a negative association at higher post-outcome uniqueness ratings. That contrast cannot be attributed to human likeness alone. Warmth, competence, realistic threat, and uniqueness threat remain plausible parts of the social judgment surrounding AI teammates. The present designs identify patterns that should guide stricter tests, not a general rule for making AI more human.

## Data Availability

The datasets presented in this article are not readily available because the dataset contains anonymized human participant responses. Access is restricted to protect participant privacy and confidentiality and will be granted only for reasonable academic purposes, subject to ethical approval and applicable institutional requirements. Requests to access the datasets should be directed to Deyu Yan, yandeyu@mail.sdu.edu.cn.

## Footnotes

1
https://aspredicted.org/d3ng-h7qz.pdf
