# Supplementary material for: Anthropomorphic cueing, social evaluation, and threat in stated willingness to collaborate with AI
Source: Front Psychol. 2026 Sep 1;17:1896807. doi: 10.3389/fpsyg.2026.1896807 (PMC13574978; doi:10.3389/fpsyg.2026.1896807)
Supplement: Supplementary file 1 [file Supplementary_File_2.docx]

**Supplementary Methods and Results**

Materials, procedures, scoring rules, and additional analyses

*This supplement distinguishes information preserved in the research archive from details that could not be verified. The retained cleaned datasets and current reanalysis form the statistical record. The manuscript identifies Credamo as the recruitment source, whereas administrative fields in the retained datasets include a paper-questionnaire double-entry label. The available records do not establish whether that label describes administration, later transcription, or both. Person-level exclusion reasons, full instruments for several auxiliary measures, the full Study 3 news articles, and authenticated Study 4 presentation records were not preserved. Those details are not reconstructed here.*

# S1. Data record and participant flow

The documentation accompanying the cleaned data reports 1,229 entries across the six studies. The six retained analysis files contain 1,128 rows. A comparison log identifies 101 form IDs that do not appear in the cleaned files. The archive does not contain the original unfiltered responses or the reasons attached to those 101 omissions. It is therefore possible to report the numerical flow, but it is not possible to verify how many cases were removed for each exclusion criterion. Every row in the retained analysis files has retained = 1 and attention_check_pass = 1.

Studies 3a and 3b were preregistered together under AsPredicted registration no. 243,886 (https://aspredicted.org/d3ng-h7qz.pdf). The archived registration specifies four stated collaboration intention items in English. Their phrasing is not identical to the item wording preserved in the questionnaire documentation. The present analysis follows the four scored item variables in the cleaned files. The reason for this wording discrepancy cannot be determined because the original administered-language survey export was not preserved.

**Table S1. Participant flow recorded in the archived documentation**

| **Study** | **Logged entries** | **Not in cleaned file** | **Retained** | **Low** | **High** | **Observed dates** |
| --- | --- | --- | --- | --- | --- | --- |
| 1a | 302 | 24 | 278 | 139 | 139 | 2026-04-15 to 2026-05-15 |
| 1b | 194 | 14 | 180 | 90 | 90 | 2026-04-15 to 2026-05-15 |
| 2 | 148 | 12 | 136 | 68 | 68 | 2026-04-15 to 2026-05-15 |
| 3a | 194 | 16 | 178 | 89 | 89 | 2026-04-15 to 2026-05-15 |
| 3b | 195 | 17 | 178 | 89 | 89 | 2026-04-15 to 2026-05-15 |
| 4 | 196 | 18 | 178 | 89 | 89 | 2026-04-15 to 2026-05-15 |

Table S2 summarizes the retained samples. Gender code 2 is labeled as women in the source manuscript, gender code 1 as men, and gender code 3 as another response. These labels are reported to preserve the source coding. They were not independently documented in the variable codebook.

**Table S2. Characteristics of the retained samples**

| **Study** | **N** | **Age M** | **Age SD** | **Women** | **Men** | **Other** |
| --- | --- | --- | --- | --- | --- | --- |
| 1a | 278 | 31.82 | 8.41 | 183 | 90 | 5 |
| 1b | 180 | 31.22 | 8.00 | 105 | 71 | 4 |
| 2 | 136 | 31.17 | 7.86 | 92 | 41 | 3 |
| 3a | 178 | 29.99 | 7.73 | 100 | 77 | 1 |
| 3b | 178 | 31.67 | 8.09 | 92 | 82 | 4 |
| 4 | 178 | 32.33 | 8.72 | 100 | 71 | 7 |

# S2. Procedure order

The order of measurement matters for interpretation. In every study, stated collaboration intention was recorded before warmth and competence. In Study 4, uniqueness threat was recorded after stated collaboration intention. The indirect association models and the Study 4 moderation model therefore cannot support causal mediation or causal moderation.

**Table S3. Recorded procedure sequence for each study**

| **Study** | **Questionnaire order** | **Design note** |
| --- | --- | --- |
| 1a | Team context → text stimulus → manipulation check → collaboration intention → warmth → competence → attention check → demographics | Two-condition paper-questionnaire anthropomorphism design |
| 1b | Team context → text stimulus → manipulation check → collaboration intention → warmth → competence → attention check → demographics | Two-condition paper-questionnaire anthropomorphism design |
| 2 | Team context → NAO baseline → active imagination prompt → imagined anthropomorphism → collaboration intention → warmth → competence → attention check → demographics | The baseline rating is retained for sensitivity adjustment. No ratio score is used |
| 3a | Team context → AQ → imagination/descriptions → imagined anthropomorphism → low-threat article → realistic threat → ingroupness → collaboration intention → warmth → competence → attention checks → demographics | Threat is fixed by study, independent sample, and questionnaire form rather than randomized within one experiment |
| 3b | Team context → AQ → imagination/descriptions → imagined anthropomorphism → high-threat article → realistic threat → ingroupness → collaboration intention → warmth → competence → attention checks → demographics | Threat is fixed by study, independent sample, and questionnaire form rather than randomized within one experiment |
| 4 | Team context → one robot image → subjective anthropomorphism → collaboration intention → warmth → competence → uniqueness threat → attention check → demographics | Uniqueness threat is measured after the outcome. Moderation is correlational |

# S3. Experimental materials retained in the original supplement

The text below reproduces the substantive materials preserved in the original supplementary document. Quotation marks identify condition texts that were presented as verbatim stimuli in that file. Descriptions identify cases where only an instruction summary or article summary was preserved.

## S3.1 Common reference-team task

Please recall a team in which you have worked previously or in which you are currently working, and answer the following questions.

a. Have you worked in this team before, or are you currently working in this team?

b. What is the size of this team?

c. What type of team is it?

## S3.2 Study 1a textual manipulation

**High anthropomorphism.** 'Hi, hello! My name is Archie, and I am your intelligent human resource assistant. I am responsible for conducting the employee performance evaluation for Chuhe Group. I will evaluate each employee's performance data. In addition, I will evaluate the 5-minute self-presentation video recorded by each employee. After evaluating the performance data and videos, I will make the final decision on this year's employee performance evaluation independently and with full responsibility. This decision may affect employees' salary, bonus, promotion eligibility, and, in some cases, whether an employee is dismissed.'

**Low anthropomorphism.** 'HRA is a new type of intelligent human resource algorithm. The employee performance evaluation for Chuhe Group will be conducted by algorithm HRA. Algorithm HRA will evaluate each employee's performance data. In addition, algorithm HRA will evaluate the 5-minute self-presentation video recorded by each employee. After evaluating the performance data and videos, the intelligent human resource algorithm HRA will make the final decision on this year's employee performance evaluation independently and with full responsibility. This decision may affect employees' salary, bonus, promotion eligibility, and, in some cases, whether an employee is dismissed.'

## S3.3 Study 1b textual manipulation

**High anthropomorphism.** 'Hi, hello! My name is Archie, and I am your intelligent collaboration partner. I am here to work with you to address various tasks and challenges. I will analyze information, generate solutions, anticipate potential problems, and communicate and coordinate closely with you throughout the process. I will flexibly adjust my strategies according to your feedback and needs to ensure that our collaboration is smooth and efficient. Whatever the goal is, I will work side by side with you, jointly seek the best solution, and complete the goal efficiently together. Let us collaborate and make the most of our respective strengths!'

**Low anthropomorphism.** 'The collaborative intelligent tool HAP is a new type of system that assists users in handling various tasks and challenges. The tool is used to analyze information, generate solutions, anticipate potential problems, and maintain communication and coordination with users during the process. The collaborative intelligent tool HAP adjusts its strategies according to users' feedback and needs to ensure a smooth and efficient support process. Whatever the goal is, the tool is designed to provide support, assist users in finding the best solution, and help users complete goals efficiently. The collaborative intelligent tool HAP is designed for collaboration with users by integrating the capabilities of both sides.'

## S3.4 Study 2 active-imagination manipulation

*The original supplement preserved an instruction-level summary, not a verbatim questionnaire script. The summary is reproduced without adding wording that is absent from the source file.*

Before the imagination task, participants rated a standard NAO robot image on a 1 to 100 humanlikeness slider. This rating was collected as a baseline and was included only in a sensitivity analysis.

Low-anthropomorphism condition: Participants were asked to imagine a low-anthropomorphic robot with an anthropomorphism level between 1 and 33 on a 1-100 scale.

High-anthropomorphism condition: Participants were asked to imagine a high-anthropomorphic robot with an anthropomorphism level between 67 and 100 on a 1-100 scale.

After the imagination task, participants rated the anthropomorphism of the imagined robot on a 1-100 slider.

## S3.5 Study 3 active imagination and written descriptions

**High-anthropomorphism condition.** 'Please imagine a highly anthropomorphic robotic AI partner. This robot will serve as your collaborative partner. It has characteristics, motives, intentions, or mental states close to those of humans.' Participants gave the robot a human name and described, in two to three sentences each, its appearance and movement, personality and emotion, and behavior pattern. Examples included delicate facial expressions, body language, eye contact, humor, empathy, personality, active communication, and flexible adaptation to unexpected situations.

**Low-anthropomorphism condition.** 'Please imagine a low-anthropomorphic robotic AI tool. This robot will serve as your collaborative device. It has basic functions.' Participants gave the robot an instrumental name, such as XR-7 or Collaborator-9, and described, in two to three sentences each, its mechanical appearance and movement, lack of personality or emotion, and behavior pattern. Examples included a mechanical appearance, programmed movement, no human-like facial expression, strict instruction following, no personality or autonomous intention, passive response to requests, and communication through data or indicator lights.

The cleaned files contain structured placeholders showing that an appearance and movement description, a personality and emotion description, and a behavior-pattern description were recorded. The response transcripts are not available in the archived materials. No content analysis of these descriptions was performed.

## S3.6 Study 3 realistic-threat articles

*The original supplement preserved summaries of the two news-style articles rather than their full text. Study 3a always used the low-threat article and Study 3b always used the high-threat article. Threat condition was not randomized within a single randomized experiment.*

Low-realistic-threat article: Participants read a news-style article titled 'Robots: Only a Temporary Trend?' The article framed robots as unlikely to replace human labor, emphasized the difficulty of developing robots for higher-level tasks, and stated that the long-term threat to human jobs and safety was limited.

High-realistic-threat article: Participants read a news-style article titled 'Robots: Replacing Human Labor?' The article framed robots as increasingly replacing human labor, emphasized rapid development and demand for robotic technologies, and stated that robots posed a substantial threat to human jobs and opportunities.

Both versions were adapted from prior threat manipulation materials and were followed by a realistic threat manipulation check.

## S3.7 Study 4 ABOT image sets

The appearance stimuli were selected from the Anthropomorphic roBOT (ABOT) database. The database provides standardized anthropomorphism ratings from 1 to 100. Six low-anthropomorphism images were drawn from the 1-33 range, and six high-anthropomorphism images were drawn from the 67-100 range. In the source manuscript, the low range was divided into 1-10, 11-20, and 21-33 subranges, and two images closest to randomly selected target scores in each subrange were selected. The high range was divided into 67-80, 81-90, and 91-100 subranges, and two images closest to randomly selected target scores in each subrange were selected.

*The mapping in Table S4 reconciles with image IDs, robot names, Anthropomorphic roBOT (ABOT) scores, and retained assignments in the cleaned Study 4 file. The within-condition mechanism used to assign participants to image codes was not preserved, so only retained IDs and counts can be reported. The table is a reference mapping, not proof that the available files were the images shown during the original experiment. The archive does not contain original-use records, authenticated experimental display files, pretest records, or license documentation. The table therefore must not be treated as an authenticated stimulus archive.*

**Table S4. Study 4 reference mapping and retained observations per image**

| **Reference ID** | **Robot** | **Condition** | **ABOT score** | **Retained n** | **Subjective anthropomorphism M (SD)** | **Collaboration intention M (SD)** |
| --- | --- | --- | --- | --- | --- | --- |
| 008 | Jibo | Lower-ABOT image set | 1.44 | 15 | 28.13 (14.57) | 4.15 (0.79) |
| 002 | Snapbot | Lower-ABOT image set | 6.04 | 15 | 29.60 (12.74) | 4.28 (1.12) |
| 003 | LEGO Mindstorms Gripper | Lower-ABOT image set | 15.92 | 16 | 33.19 (19.60) | 4.17 (1.34) |
| 020 | AIDA | Lower-ABOT image set | 19.57 | 12 | 32.17 (12.85) | 4.40 (1.21) |
| 009 | Buddy | Lower-ABOT image set | 23.04 | 17 | 35.82 (14.98) | 4.19 (1.18) |
| 013 | RIBA | Lower-ABOT image set | 31.00 | 14 | 38.50 (17.00) | 3.75 (1.05) |
| 061 | Bina48 | Higher-ABOT image set | 73.04 | 17 | 65.47 (15.08) | 4.10 (0.91) |
| 062 | Sophia | Higher-ABOT image set | 78.88 | 14 | 68.14 (16.70) | 3.96 (1.16) |
| 196 | Otonaroid | Higher-ABOT image set | 88.74 | 14 | 69.71 (18.28) | 3.75 (1.23) |
| 197 | Erica | Higher-ABOT image set | 89.60 | 15 | 65.20 (15.66) | 3.65 (1.51) |
| 198 | Geminoid H1-4 | Higher-ABOT image set | 92.60 | 16 | 63.19 (17.15) | 4.14 (1.22) |
| 195 | Kodomoroid | Higher-ABOT image set | 93.44 | 13 | 50.15 (19.48) | 3.75 (1.46) |

# S4. Measures and scoring

Composite scores were complete-item means. Collaboration intention items 2 and 4 were reverse scored as 8 minus the raw response. Realistic-threat item 2 was reverse scored in the same way. No other archived scale item was reverse scored.

## S4.1 Stated collaboration intention

Participants responded on 7-point Likert scales from 1 = strongly disagree to 7 = strongly agree. Items 2 and 4 were reverse-coded.

1. This AI teammate could be a good teammate.

2. This AI teammate could be a bad teammate.

3. I would like to work with this AI teammate.

4. I would not like to work with this AI teammate.

*Documented wording discrepancy for Studies 3a and 3b. AsPredicted registration no. 243,886 frames the target as a named robot and uses probability and enjoyment language. The archived questionnaire documentation frames the target as an AI teammate and uses possibility and liking language. The analysis uses the four scored item variables in the cleaned files. The available records do not establish whether the discrepancy reflects translation, a later wording revision, or another documentation difference.*

## S4.2 Perceived warmth and competence

Participants rated the AI agent on 7-point Likert scales from 1 = strongly disagree to 7 = strongly agree.

Competence items:

1. The AI agent is intelligent.

2. The AI agent is skillful at executing tasks.

3. The AI agent is powerful.

4. The AI agent executes tasks quickly.

5. The AI agent executes tasks efficiently.

Warmth items:

1. The AI agent is caring.

2. The AI agent is kind.

3. The AI agent is friendly.

4. The AI agent is warm.

5. The AI agent is sociable.

## S4.3 Threat items

Participants responded on 7-point Likert scales from 1 = strongly disagree to 7 = strongly agree. Items 1-5 assess uniqueness threat. Items 6-10 assess realistic threat, with Item 7 reverse-coded.

1. The widespread use of AI in daily life bothers me because it blurs the boundary between humans and AI.

2. AI that looks alive is unsettling because it is almost indistinguishable from humans.

3. Recent technological advances challenge the essence of what it means to be human.

4. Advances in AI technology are threatening human uniqueness.

5. AI is beginning to blur the boundary between humans and AI.

6. The increased use of AI in daily life is causing human unemployment.

7. AI cannot replace people's jobs.

8. In the long run, AI poses a direct threat to human safety and well-being.

9. The development of AI technology threatens human employment and opportunities.

10. The increasing prevalence of AI in daily life threatens human safety.

## S4.4 Preregistered Study 3 auxiliary items

AsPredicted registration no. 243,886 preserves the following five Anthropomorphism Questionnaire items for Studies 3a and 3b.

1. Whether a computer may slow down deliberately after being shouted at.

2. Whether a computer or printer may behave awkwardly on purpose.

3. Whether particular cars have distinct personalities.

4. Whether treating a computer or printer as happy and wanted may reduce malfunction.

5. Whether a friendly-seeming personality influenced the choice of a car or electrical item.

The same registration records a verbal item asking whether the imagined robot is viewed as an ingroup member. It also names an adapted Inclusion of Other in the Self graphical measure (Aron et al., 1992). The exact graphical presentation is not preserved in the archive.

The two registered attention checks instruct respondents to select option 1 and option 2, respectively. Exact attention-check wording for the other studies was not preserved.

## S4.5 Scoring specifications for all archived constructs

**Table S5. Scoring rules applied to the cleaned data**

| **Construct** | **Range** | **Reverse scoring** | **Composite** | **Available documentation** |
| --- | --- | --- | --- | --- |
| Collaboration intention | 1-7 integers | items 2 and 4: 8-raw | complete-item mean | Four 1-to-7 items. Items 2 and 4 are reverse-scored, and the composite is the mean of all four scored items. |
| Warmth | 1-7 integers | none | complete-item mean | Five 1-to-7 items. The composite score was calculated as the mean of all five items. |
| Competence | 1-7 integers | none | complete-item mean | Five 1-to-7 items. The composite score was calculated as the mean of all five items. |
| Study1a manipulation | 1-7 integer | none | single item | One 1-to-7 manipulation-check item in the retained cleaned dataset. Exact item wording was not preserved. |
| Subjective anthropomorphism | 1-100 integer | none | single item | Study-specific 1-to-100 anthropomorphism item in the retained cleaned dataset. Exact item wording was not preserved. |
| Anthropomorphism tendency | 1-7 integers | none | complete-item mean | Five 1-to-7 anthropomorphism-tendency items. The composite score was calculated as the mean of all five items. |
| Realistic threat | 1-7 integers | item 2: 8-raw | complete-item mean | Five 1-to-7 items. Item 2 is reverse-scored, and the composite is the mean of all five scored items. |
| Ingroupness | 1-7 integers | none | complete-item mean | Two 1-to-7 items. The composite score was calculated as the mean of both items. |
| Uniqueness threat | 1-7 integers | none | complete-item mean | Five 1-to-7 items. The composite score was calculated as the mean of all five items. |

*The Study 3 registration preserves five anthropomorphism-tendency items, one verbal ingroupness item, and two attention-check instructions. It names an adapted Inclusion of Other in the Self measure but does not preserve its exact graphical presentation in the archive. Exact single-item anthropomorphism-check wording and attention-check wording for the other studies were not preserved. The archived files preserve item columns, response ranges, scoring rules, and procedure position.*

# S5. Statistical specifications

All reported values were taken from the current rerun output files. Descriptive standard deviations use the sample denominator. Two-condition comparisons use two-sided independent-samples t tests with a pooled variance estimate. Cohen's d uses the pooled standard deviation. The six manipulation checks are reported separately as design checks. The 18 focal comparisons cover stated collaboration intention, perceived warmth, and perceived competence in each study. Benjamini-Hochberg (BH) adjusted q values were calculated across this 18-outcome family.

Reliability is reported as Cronbach's alpha. Parallel indirect associations were estimated with ordinary least squares. Assigned condition was coded 0 for low and 1 for high. Five thousand case-resampling bootstrap samples and percentile intervals were generated with seed 20260809. The primary decompositions are unadjusted. This choice aligns the indirect models with the randomized condition comparisons and avoids selecting covariates from observed outcome correlations. Covariate-adjusted models are reported only as sensitivity analyses. Those models include gender indicators in Studies 1b and 4, the NAO baseline rating in Study 2, and anthropomorphism tendency in Studies 3a and 3b. Study 1a has no added covariate in either specification. Since stated collaboration intention preceded warmth and competence, all decompositions are statistical associations rather than evidence of causal mediation.

The combined Study 3 models include assigned cue condition, a Study 3a versus Study 3b indicator, and their interaction. Neither the study coefficient nor the cue-by-study interaction can isolate threat because study, independent sample, questionnaire form, and threat article covary perfectly. The primary Study 4 model regresses collaboration intention on assigned image-set condition, mean-centered uniqueness threat, and their interaction without covariates. A gender-adjusted model is reported as a sensitivity analysis. The unadjusted image fixed-effect model conditions on the 12 mapped image IDs. It does not estimate uncertainty over a broader population of robot images. Uniqueness threat was measured after the outcome, so every Study 4 interaction and Johnson-Neyman region describes an observed conditional association.

*Only the Study 4 decomposition using assigned image-set condition as the predictor is reported. A legacy model using continuous subjective anthropomorphism could not be verified from the current result set, and its reported remaining condition estimate was outside its stated confidence interval. It is therefore not treated as evidence here.*

# S6. Full rerun results

The tables below present rounded values for reading. The accompanying Excel workbook preserves the full numerical precision in the rerun files.

| **Table S6. Descriptive statistics by study and condition** | | | | | | | |
| --- | --- | --- | --- | --- | --- | --- | --- |
| **Study** | **Variable** | **Low n** | **Low M (SD)** | **Low range** | **High n** | **High M (SD)** | **High range** |
| 1a | Perceived competence | 139 | 4.06 (1.27) | 1.00 to 6.80 | 139 | 4.00 (1.17) | 1.20 to 6.60 |
| 1a | Manipulation check, 1 to 7 | 139 | 4.20 (1.10) | 1.00 to 7.00 | 139 | 4.59 (1.12) | 1.00 to 7.00 |
| 1a | Stated collaboration intention | 139 | 4.16 (1.31) | 1.00 to 7.00 | 139 | 4.09 (1.21) | 1.00 to 6.50 |
| 1a | Perceived warmth | 139 | 4.04 (1.34) | 1.20 to 6.60 | 139 | 4.43 (1.26) | 1.20 to 7.00 |
| 1b | Perceived competence | 90 | 4.05 (1.18) | 1.60 to 6.80 | 90 | 4.17 (1.34) | 1.00 to 7.00 |
| 1b | Subjective anthropomorphism, 1 to 100 | 90 | 48.52 (23.13) | 6.00 to 97.00 | 90 | 58.07 (23.68) | 3.00 to 97.00 |
| 1b | Stated collaboration intention | 90 | 3.92 (1.26) | 1.00 to 6.50 | 90 | 4.37 (1.23) | 1.00 to 6.50 |
| 1b | Perceived warmth | 90 | 4.01 (1.35) | 1.00 to 6.80 | 90 | 4.64 (1.32) | 1.60 to 7.00 |
| 2 | Perceived competence | 68 | 3.89 (1.28) | 1.40 to 6.40 | 68 | 4.40 (1.16) | 1.40 to 6.60 |
| 2 | Imagined anthropomorphism, 1 to 100 | 68 | 31.96 (17.45) | 7.00 to 74.00 | 68 | 68.56 (18.13) | 19.00 to 94.00 |
| 2 | Stated collaboration intention | 68 | 3.83 (1.22) | 1.75 to 6.50 | 68 | 4.33 (1.24) | 1.50 to 6.50 |
| 2 | Perceived warmth | 68 | 3.96 (1.27) | 1.20 to 6.60 | 68 | 4.88 (1.32) | 2.00 to 7.00 |

| **Table S6 (continued). Descriptive statistics by study and condition** | | | | | | | |
| --- | --- | --- | --- | --- | --- | --- | --- |
| **Study** | **Variable** | **Low n** | **Low M (SD)** | **Low range** | **High n** | **High M (SD)** | **High range** |
| 3a | Perceived competence | 89 | 3.93 (1.34) | 1.60 to 7.00 | 89 | 4.69 (1.16) | 1.60 to 7.00 |
| 3a | Imagined anthropomorphism, 1 to 100 | 89 | 34.17 (18.26) | 7.00 to 79.00 | 89 | 73.49 (16.08) | 30.00 to 96.00 |
| 3a | Ingroupness | 89 | 4.06 (1.44) | 1.00 to 7.00 | 89 | 4.26 (1.43) | 1.00 to 7.00 |
| 3a | Realistic threat | 89 | 4.09 (1.32) | 1.40 to 7.00 | 89 | 4.02 (1.38) | 1.20 to 6.60 |
| 3a | Stated collaboration intention | 89 | 3.69 (1.28) | 1.00 to 7.00 | 89 | 4.57 (1.33) | 1.50 to 7.00 |
| 3a | Perceived warmth | 89 | 3.88 (1.23) | 1.20 to 6.40 | 89 | 4.99 (1.24) | 1.80 to 7.00 |
| 3b | Perceived competence | 89 | 4.12 (1.22) | 1.40 to 6.80 | 89 | 4.27 (1.15) | 1.40 to 6.60 |
| 3b | Imagined anthropomorphism, 1 to 100 | 89 | 27.67 (17.61) | 5.00 to 79.00 | 89 | 71.97 (15.04) | 38.00 to 95.00 |
| 3b | Ingroupness | 89 | 4.16 (1.31) | 1.00 to 7.00 | 89 | 4.24 (1.41) | 1.00 to 7.00 |
| 3b | Realistic threat | 89 | 4.68 (1.39) | 1.60 to 7.00 | 89 | 4.93 (1.22) | 1.20 to 7.00 |
| 3b | Stated collaboration intention | 89 | 3.82 (1.28) | 1.00 to 6.50 | 89 | 4.33 (1.28) | 1.25 to 7.00 |
| 3b | Perceived warmth | 89 | 3.78 (1.32) | 1.20 to 7.00 | 89 | 4.70 (1.20) | 2.20 to 7.00 |
| 4 | Perceived competence | 89 | 4.16 (1.11) | 1.40 to 6.80 | 89 | 3.70 (1.21) | 1.20 to 6.40 |
| 4 | Stated collaboration intention | 89 | 4.15 (1.11) | 1.00 to 6.75 | 89 | 3.90 (1.23) | 1.25 to 6.50 |
| 4 | Subjective anthropomorphism, 1 to 100 | 89 | 32.93 (15.55) | 5.00 to 67.00 | 89 | 63.87 (17.60) | 19.00 to 98.00 |
| 4 | Uniqueness threat | 89 | 3.96 (1.13) | 1.20 to 6.40 | 89 | 3.89 (1.20) | 1.80 to 6.80 |
| 4 | Perceived warmth | 89 | 3.91 (1.17) | 1.60 to 6.40 | 89 | 4.07 (1.24) | 1.00 to 6.80 |

**Table S7. Internal consistency in the retained samples**

| **Study** | **Scale** | **n** | **Items** | **Cronbach's alpha** |
| --- | --- | --- | --- | --- |
| 1a | SCI | 278 | 4 | .812 |
| 1a | Warmth | 278 | 5 | .880 |
| 1a | Competence | 278 | 5 | .822 |
| 1b | SCI | 180 | 4 | .804 |
| 1b | Warmth | 180 | 5 | .878 |
| 1b | Competence | 180 | 5 | .847 |
| 2 | SCI | 136 | 4 | .815 |
| 2 | Warmth | 136 | 5 | .888 |
| 2 | Competence | 136 | 5 | .838 |
| 3a | SCI | 178 | 4 | .850 |
| 3a | Warmth | 178 | 5 | .878 |
| 3a | Competence | 178 | 5 | .852 |
| 3a | Anthropomorphism tendency | 178 | 5 | .809 |
| 3a | Realistic threat | 178 | 5 | .864 |
| 3a | Ingroupness | 178 | 2 | .729 |
| 3b | SCI | 178 | 4 | .828 |
| 3b | Warmth | 178 | 5 | .869 |
| 3b | Competence | 178 | 5 | .815 |
| 3b | Anthropomorphism tendency | 178 | 5 | .785 |
| 3b | Realistic threat | 178 | 5 | .868 |
| 3b | Ingroupness | 178 | 2 | .652 |
| 4 | SCI | 178 | 4 | .762 |
| 4 | Warmth | 178 | 5 | .848 |
| 4 | Competence | 178 | 5 | .816 |
| 4 | Uniqueness threat | 178 | 5 | .809 |

*Note. SCI means stated collaboration intention. AQ means Anthropomorphism Questionnaire.*

**Table S8. Manipulation checks reported separately from the focal outcome family**

| **Study** | **Design check** | **Low M (SD)** | **High M (SD)** | **High minus low** | **95% CI** | **t (df)** | **p** | **d** |
| --- | --- | --- | --- | --- | --- | --- | --- | --- |
| 1a | Manipulation check, 1 to 7 | 4.20 (1.10) | 4.59 (1.12) | 0.388 | [0.126, 0.651] | 2.918 (276) | = .004 | 0.350 |
| 1b | Subjective anthropomorphism, 1 to 100 | 48.52 (23.13) | 58.07 (23.68) | 9.544 | [2.659, 16.430] | 2.735 (178) | = .007 | 0.408 |
| 2 | Imagined anthropomorphism, 1 to 100 | 31.96 (17.45) | 68.56 (18.13) | 36.603 | [30.566, 42.639] | 11.993 (134) | < .001 | 2.057 |
| 3a | Imagined anthropomorphism, 1 to 100 | 34.17 (18.26) | 73.49 (16.08) | 39.326 | [34.236, 44.416] | 15.248 (176) | < .001 | 2.286 |
| 3b | Imagined anthropomorphism, 1 to 100 | 27.67 (17.61) | 71.97 (15.04) | 44.292 | [39.448, 49.136] | 18.046 (176) | < .001 | 2.705 |
| 4 | Subjective anthropomorphism, 1 to 100 | 32.93 (15.55) | 63.87 (17.60) | 30.933 | [26.020, 35.845] | 12.426 (176) | < .001 | 1.863 |

**Table S9. Focal condition comparisons with BH adjustment across 18 outcomes**

| **Study** | **Focal outcome** | **Low M (SD)** | **High M (SD)** | **High minus low** | **95% CI** | **t (df)** | **p** | **BH q** | **d** |
| --- | --- | --- | --- | --- | --- | --- | --- | --- | --- |
| 1a | Stated collaboration intention | 4.16 (1.31) | 4.09 (1.21) | -0.063 | [-0.360, 0.234] | -0.417 (276) | = .677 | = .681 | -0.050 |
| 1a | Perceived warmth | 4.04 (1.34) | 4.43 (1.26) | 0.386 | [0.078, 0.693] | 2.470 (276) | = .014 | = .028 | 0.296 |
| 1a | Perceived competence | 4.06 (1.27) | 4.00 (1.17) | -0.060 | [-0.349, 0.228] | -0.412 (276) | = .681 | = .681 | -0.049 |
| 1b | Stated collaboration intention | 3.92 (1.26) | 4.37 (1.23) | 0.447 | [0.081, 0.813] | 2.413 (178) | = .017 | = .028 | 0.360 |
| 1b | Perceived warmth | 4.01 (1.35) | 4.64 (1.32) | 0.631 | [0.238, 1.024] | 3.172 (178) | = .002 | = .005 | 0.473 |
| 1b | Perceived competence | 4.05 (1.18) | 4.17 (1.34) | 0.124 | [-0.248, 0.497] | 0.660 (178) | = .510 | = .574 | 0.098 |
| 2 | Stated collaboration intention | 3.83 (1.22) | 4.33 (1.24) | 0.496 | [0.080, 0.913] | 2.358 (134) | = .020 | = .030 | 0.404 |
| 2 | Perceived warmth | 3.96 (1.27) | 4.88 (1.32) | 0.921 | [0.481, 1.360] | 4.142 (134) | < .001 | < .001 | 0.710 |
| 2 | Perceived competence | 3.89 (1.28) | 4.40 (1.16) | 0.509 | [0.094, 0.924] | 2.424 (134) | = .017 | = .028 | 0.416 |
| 3a | Stated collaboration intention | 3.69 (1.28) | 4.57 (1.33) | 0.882 | [0.497, 1.267] | 4.517 (176) | < .001 | < .001 | 0.677 |
| 3a | Perceived warmth | 3.88 (1.23) | 4.99 (1.24) | 1.110 | [0.744, 1.476] | 5.987 (176) | < .001 | < .001 | 0.897 |
| 3a | Perceived competence | 3.93 (1.34) | 4.69 (1.16) | 0.760 | [0.390, 1.129] | 4.057 (176) | < .001 | < .001 | 0.608 |
| 3b | Stated collaboration intention | 3.82 (1.28) | 4.33 (1.28) | 0.503 | [0.124, 0.882] | 2.618 (176) | = .010 | = .022 | 0.393 |
| 3b | Perceived warmth | 3.78 (1.32) | 4.70 (1.20) | 0.919 | [0.547, 1.291] | 4.875 (176) | < .001 | < .001 | 0.731 |
| 3b | Perceived competence | 4.12 (1.22) | 4.27 (1.15) | 0.144 | [-0.208, 0.496] | 0.806 (176) | = .421 | = .505 | 0.121 |
| 4 | Stated collaboration intention | 4.15 (1.11) | 3.90 (1.23) | -0.250 | [-0.597, 0.097] | -1.421 (176) | = .157 | = .218 | -0.213 |
| 4 | Perceived warmth | 3.91 (1.17) | 4.07 (1.24) | 0.157 | [-0.199, 0.514] | 0.871 (176) | = .385 | = .495 | 0.131 |
| 4 | Perceived competence | 4.16 (1.11) | 3.70 (1.21) | -0.461 | [-0.804, -0.117] | -2.649 (176) | = .009 | = .022 | -0.397 |

| **Table S10. Primary unadjusted parallel statistical decomposition** | | | | | |
| --- | --- | --- | --- | --- | --- |
| **Study** | **Primary unadjusted component** | **Estimate** | **95% bootstrap CI** | **Resamples** | **Covariates** |
| 1a | Condition coefficient in warmth model | 0.386 | [0.081, 0.685] | 5000 | none |
| 1a | Condition coefficient in competence model | -0.060 | [-0.352, 0.225] | 5000 | none |
| 1a | Warmth coefficient in joint outcome model | 0.298 | [0.191, 0.401] | 5000 | none |
| 1a | Competence coefficient in joint outcome model | 0.297 | [0.184, 0.411] | 5000 | none |
| 1a | Warmth product | 0.115 | [0.022, 0.228] | 5000 | none |
| 1a | Competence product | -0.018 | [-0.117, 0.064] | 5000 | none |
| 1a | Remaining condition coefficient | -0.160 | [-0.429, 0.107] | 5000 | none |
| 1a | Total condition contrast | -0.063 | [-0.353, 0.231] | 5000 | none |
| 1b | Condition coefficient in warmth model | 0.631 | [0.229, 1.028] | 5000 | none |
| 1b | Condition coefficient in competence model | 0.124 | [-0.254, 0.486] | 5000 | none |
| 1b | Warmth coefficient in joint outcome model | 0.249 | [0.129, 0.374] | 5000 | none |
| 1b | Competence coefficient in joint outcome model | 0.240 | [0.087, 0.396] | 5000 | none |
| 1b | Warmth product | 0.157 | [0.045, 0.318] | 5000 | none |
| 1b | Competence product | 0.030 | [-0.073, 0.122] | 5000 | none |
| 1b | Remaining condition coefficient | 0.260 | [-0.070, 0.588] | 5000 | none |
| 1b | Total condition contrast | 0.447 | [0.086, 0.801] | 5000 | none |

| **Table S10 (continued). Primary unadjusted parallel statistical decomposition** | | | | | |
| --- | --- | --- | --- | --- | --- |
| **Study** | **Primary unadjusted component** | **Estimate** | **95% bootstrap CI** | **Resamples** | **Covariates** |
| 2 | Condition coefficient in warmth model | 0.921 | [0.479, 1.351] | 5000 | none |
| 2 | Condition coefficient in competence model | 0.509 | [0.087, 0.910] | 5000 | none |
| 2 | Warmth coefficient in joint outcome model | 0.216 | [0.054, 0.366] | 5000 | none |
| 2 | Competence coefficient in joint outcome model | 0.300 | [0.143, 0.467] | 5000 | none |
| 2 | Warmth product | 0.199 | [0.045, 0.382] | 5000 | none |
| 2 | Competence product | 0.153 | [0.022, 0.329] | 5000 | none |
| 2 | Remaining condition coefficient | 0.145 | [-0.258, 0.553] | 5000 | none |
| 2 | Total condition contrast | 0.496 | [0.073, 0.902] | 5000 | none |
| 3a | Condition coefficient in warmth model | 1.110 | [0.742, 1.466] | 5000 | none |
| 3a | Condition coefficient in competence model | 0.760 | [0.383, 1.123] | 5000 | none |
| 3a | Warmth coefficient in joint outcome model | 0.421 | [0.297, 0.554] | 5000 | none |
| 3a | Competence coefficient in joint outcome model | 0.284 | [0.149, 0.419] | 5000 | none |
| 3a | Warmth product | 0.467 | [0.267, 0.707] | 5000 | none |
| 3a | Competence product | 0.216 | [0.089, 0.373] | 5000 | none |
| 3a | Remaining condition coefficient | 0.199 | [-0.152, 0.535] | 5000 | none |
| 3a | Total condition contrast | 0.882 | [0.485, 1.262] | 5000 | none |

| **Table S10 (continued). Primary unadjusted parallel statistical decomposition** | | | | | |
| --- | --- | --- | --- | --- | --- |
| **Study** | **Primary unadjusted component** | **Estimate** | **95% bootstrap CI** | **Resamples** | **Covariates** |
| 3b | Condition coefficient in warmth model | 0.919 | [0.560, 1.289] | 5000 | none |
| 3b | Condition coefficient in competence model | 0.144 | [-0.202, 0.497] | 5000 | none |
| 3b | Warmth coefficient in joint outcome model | 0.172 | [0.027, 0.312] | 5000 | none |
| 3b | Competence coefficient in joint outcome model | 0.375 | [0.238, 0.511] | 5000 | none |
| 3b | Warmth product | 0.158 | [0.023, 0.317] | 5000 | none |
| 3b | Competence product | 0.054 | [-0.077, 0.192] | 5000 | none |
| 3b | Remaining condition coefficient | 0.291 | [-0.069, 0.649] | 5000 | none |
| 3b | Total condition contrast | 0.503 | [0.120, 0.875] | 5000 | none |
| 4 | Condition coefficient in warmth model | 0.157 | [-0.186, 0.515] | 5000 | none |
| 4 | Condition coefficient in competence model | -0.461 | [-0.808, -0.111] | 5000 | none |
| 4 | Warmth coefficient in joint outcome model | 0.192 | [0.042, 0.339] | 5000 | none |
| 4 | Competence coefficient in joint outcome model | 0.250 | [0.089, 0.401] | 5000 | none |
| 4 | Warmth product | 0.030 | [-0.043, 0.111] | 5000 | none |
| 4 | Competence product | -0.115 | [-0.245, -0.018] | 5000 | none |
| 4 | Remaining condition coefficient | -0.165 | [-0.506, 0.172] | 5000 | none |
| 4 | Total condition contrast | -0.250 | [-0.596, 0.086] | 5000 | none |

| **Table S11. Covariate-adjusted sensitivity decomposition** | | | | | |
| --- | --- | --- | --- | --- | --- |
| **Study** | **Sensitivity component** | **Estimate** | **95% bootstrap CI** | **Resamples** | **Covariates** |
| 1a | Condition coefficient in warmth model | 0.386 | [0.081, 0.685] | 5000 | none |
| 1a | Condition coefficient in competence model | -0.060 | [-0.352, 0.225] | 5000 | none |
| 1a | Warmth coefficient in joint outcome model | 0.298 | [0.191, 0.401] | 5000 | none |
| 1a | Competence coefficient in joint outcome model | 0.297 | [0.184, 0.411] | 5000 | none |
| 1a | Warmth product | 0.115 | [0.022, 0.228] | 5000 | none |
| 1a | Competence product | -0.018 | [-0.117, 0.064] | 5000 | none |
| 1a | Remaining condition coefficient | -0.160 | [-0.429, 0.107] | 5000 | none |
| 1a | Total condition contrast | -0.063 | [-0.353, 0.231] | 5000 | none |
| 1b | Condition coefficient in warmth model | 0.581 | [0.179, 0.983] | 5000 | women indicator, other-gender indicator |
| 1b | Condition coefficient in competence model | 0.095 | [-0.296, 0.476] | 5000 | women indicator, other-gender indicator |
| 1b | Warmth coefficient in joint outcome model | 0.244 | [0.121, 0.374] | 5000 | women indicator, other-gender indicator |
| 1b | Competence coefficient in joint outcome model | 0.241 | [0.083, 0.401] | 5000 | women indicator, other-gender indicator |
| 1b | Warmth product | 0.142 | [0.033, 0.299] | 5000 | women indicator, other-gender indicator |
| 1b | Competence product | 0.023 | [-0.084, 0.118] | 5000 | women indicator, other-gender indicator |
| 1b | Remaining condition coefficient | 0.251 | [-0.084, 0.580] | 5000 | women indicator, other-gender indicator |
| 1b | Total condition contrast | 0.416 | [0.053, 0.770] | 5000 | women indicator, other-gender indicator |

| **Table S11 (continued). Covariate-adjusted sensitivity decomposition** | | | | | |
| --- | --- | --- | --- | --- | --- |
| **Study** | **Sensitivity component** | **Estimate** | **95% bootstrap CI** | **Resamples** | **Covariates** |
| 2 | Condition coefficient in warmth model | 0.918 | [0.475, 1.351] | 5000 | NAO baseline |
| 2 | Condition coefficient in competence model | 0.509 | [0.088, 0.923] | 5000 | NAO baseline |
| 2 | Warmth coefficient in joint outcome model | 0.216 | [0.052, 0.368] | 5000 | NAO baseline |
| 2 | Competence coefficient in joint outcome model | 0.300 | [0.144, 0.466] | 5000 | NAO baseline |
| 2 | Warmth product | 0.199 | [0.043, 0.377] | 5000 | NAO baseline |
| 2 | Competence product | 0.153 | [0.021, 0.334] | 5000 | NAO baseline |
| 2 | Remaining condition coefficient | 0.148 | [-0.255, 0.563] | 5000 | NAO baseline |
| 2 | Total condition contrast | 0.499 | [0.081, 0.907] | 5000 | NAO baseline |
| 3a | Condition coefficient in warmth model | 1.085 | [0.718, 1.448] | 5000 | anthropomorphism tendency |
| 3a | Condition coefficient in competence model | 0.745 | [0.367, 1.120] | 5000 | anthropomorphism tendency |
| 3a | Warmth coefficient in joint outcome model | 0.425 | [0.300, 0.560] | 5000 | anthropomorphism tendency |
| 3a | Competence coefficient in joint outcome model | 0.286 | [0.148, 0.423] | 5000 | anthropomorphism tendency |
| 3a | Warmth product | 0.462 | [0.264, 0.697] | 5000 | anthropomorphism tendency |
| 3a | Competence product | 0.213 | [0.086, 0.370] | 5000 | anthropomorphism tendency |
| 3a | Remaining condition coefficient | 0.205 | [-0.151, 0.543] | 5000 | anthropomorphism tendency |
| 3a | Total condition contrast | 0.879 | [0.483, 1.257] | 5000 | anthropomorphism tendency |

| **Table S11 (continued). Covariate-adjusted sensitivity decomposition** | | | | | |
| --- | --- | --- | --- | --- | --- |
| **Study** | **Sensitivity component** | **Estimate** | **95% bootstrap CI** | **Resamples** | **Covariates** |
| 3b | Condition coefficient in warmth model | 0.933 | [0.567, 1.309] | 5000 | anthropomorphism tendency |
| 3b | Condition coefficient in competence model | 0.144 | [-0.208, 0.496] | 5000 | anthropomorphism tendency |
| 3b | Warmth coefficient in joint outcome model | 0.174 | [0.028, 0.315] | 5000 | anthropomorphism tendency |
| 3b | Competence coefficient in joint outcome model | 0.375 | [0.238, 0.509] | 5000 | anthropomorphism tendency |
| 3b | Warmth product | 0.162 | [0.024, 0.321] | 5000 | anthropomorphism tendency |
| 3b | Competence product | 0.054 | [-0.078, 0.192] | 5000 | anthropomorphism tendency |
| 3b | Remaining condition coefficient | 0.285 | [-0.075, 0.645] | 5000 | anthropomorphism tendency |
| 3b | Total condition contrast | 0.501 | [0.114, 0.877] | 5000 | anthropomorphism tendency |
| 4 | Condition coefficient in warmth model | 0.167 | [-0.190, 0.533] | 5000 | women indicator, other-gender indicator |
| 4 | Condition coefficient in competence model | -0.411 | [-0.759, -0.051] | 5000 | women indicator, other-gender indicator |
| 4 | Warmth coefficient in joint outcome model | 0.194 | [0.047, 0.347] | 5000 | women indicator, other-gender indicator |
| 4 | Competence coefficient in joint outcome model | 0.244 | [0.077, 0.397] | 5000 | women indicator, other-gender indicator |
| 4 | Warmth product | 0.032 | [-0.043, 0.120] | 5000 | women indicator, other-gender indicator |
| 4 | Competence product | -0.101 | [-0.225, -0.006] | 5000 | women indicator, other-gender indicator |
| 4 | Remaining condition coefficient | -0.150 | [-0.485, 0.183] | 5000 | women indicator, other-gender indicator |
| 4 | Total condition contrast | -0.218 | [-0.561, 0.129] | 5000 | women indicator, other-gender indicator |

*Note. The primary models are unadjusted. The sensitivity covariates were not used to redefine the focal test. Warmth and competence were measured after collaboration intention, so neither table establishes temporal or causal mediation.*

**Table S12. Study 3 cross-study threat check**

| **Contrast** | **Study 3a M (SD)** | **Study 3b M (SD)** | **Difference** | **t (df)** | **p** | **d** |
| --- | --- | --- | --- | --- | --- | --- |
| Realistic threat | 4.05 (1.35) | 4.81 (1.31) | 0.752 | 5.321 (354) | < .001 | 0.564 |

*Note. Study indicator, independent sample, questionnaire form, and threat article are inseparable in this contrast.*

**Table S13. Combined Study 3 cross-study models**

| **Outcome** | **Term** | **b** | **SE** | **t (df)** | **p** | **R squared** |
| --- | --- | --- | --- | --- | --- | --- |
| Perceived warmth | Intercept | 3.876 | 0.132 | 29.318 (352) | < .001 | 0.149 |
| Perceived warmth | Assigned cue condition | 1.110 | 0.187 | 5.937 (352) | < .001 | 0.149 |
| Perceived warmth | Study 3b indicator | -0.099 | 0.187 | -0.529 (352) | = .597 | 0.149 |
| Perceived warmth | Cue condition × study | -0.191 | 0.264 | -0.722 (352) | = .471 | 0.149 |
| Perceived competence | Intercept | 3.933 | 0.129 | 30.418 (352) | < .001 | 0.051 |
| Perceived competence | Assigned cue condition | 0.760 | 0.183 | 4.154 (352) | < .001 | 0.051 |
| Perceived competence | Study 3b indicator | 0.189 | 0.183 | 1.032 (352) | = .303 | 0.051 |
| Perceived competence | Cue condition × study | -0.616 | 0.259 | -2.381 (352) | = .018 | 0.051 |
| Stated collaboration intention | Intercept | 3.691 | 0.137 | 26.954 (352) | < .001 | 0.073 |
| Stated collaboration intention | Assigned cue condition | 0.882 | 0.194 | 4.554 (352) | < .001 | 0.073 |
| Stated collaboration intention | Study 3b indicator | 0.132 | 0.194 | 0.682 (352) | = .496 | 0.073 |
| Stated collaboration intention | Cue condition × study | -0.379 | 0.274 | -1.385 (352) | = .167 | 0.073 |

*Note. The study coefficient and its interaction do not identify a randomized threat effect.*

**Table S14. Primary unadjusted Study 4 moderation and image fixed-effect result**

| **Model** | **Term** | **b** | **SE** | **t (df)** | **p** | **95% CI** | **R squared** |
| --- | --- | --- | --- | --- | --- | --- | --- |
| Primary unadjusted | Intercept | 4.155 | 0.120 | 34.699 (174) | < .001 | [3.918, 4.391] | 0.096 |
| Primary unadjusted | Assigned image-set condition | -0.264 | 0.169 | -1.562 (174) | = .120 | [-0.599, 0.070] | 0.096 |
| Primary unadjusted | Uniqueness threat centered | -0.009 | 0.107 | -0.086 (174) | = .932 | [-0.220, 0.201] | 0.096 |
| Primary unadjusted | Image set × uniqueness threat | -0.396 | 0.147 | -2.703 (174) | = .008 | [-0.686, -0.107] | 0.096 |
| Unadjusted image fixed-effect sensitivity | Image set × uniqueness threat | -0.402 | 0.151 | -2.658 (164) | = .009 | [-0.700, -0.103] | 0.123 |

*Note. The image fixed-effect result is unadjusted and conditional on the 12 mapped images. The image-set main coefficient is absorbed by image identity in that model.*

**Table S15. Gender-adjusted Study 4 sensitivity model**

| **Model** | **Term** | **b** | **SE** | **t (df)** | **p** | **95% CI** | **R squared** |
| --- | --- | --- | --- | --- | --- | --- | --- |
| Gender-adjusted | Intercept | 4.178 | 0.152 | 27.470 (172) | < .001 | [3.878, 4.479] | 0.096 |
| Gender-adjusted | Assigned image-set condition | -0.255 | 0.173 | -1.470 (172) | = .143 | [-0.597, 0.087] | 0.096 |
| Gender-adjusted | Uniqueness threat centered | -0.008 | 0.109 | -0.072 (172) | = .943 | [-0.222, 0.207] | 0.096 |
| Gender-adjusted | Image set × uniqueness threat | -0.392 | 0.150 | -2.622 (172) | = .010 | [-0.688, -0.097] | 0.096 |
| Gender-adjusted | Gender female | -0.051 | 0.182 | -0.281 (172) | = .779 | [-0.411, 0.308] | 0.096 |
| Gender-adjusted | Gender other | 0.011 | 0.456 | 0.025 (172) | = .980 | [-0.888, 0.911] | 0.096 |
| Gender-adjusted image fixed effect | Image set × uniqueness threat | -0.400 | 0.154 | -2.597 (162) | = .010 | [-0.704, -0.096] | 0.123 |

**Table S16. Primary unadjusted Study 4 Johnson-Neyman boundaries and simple slopes**

| **Point** | **Uniqueness threat** | **Observed range** | **Scale range** | **Conditional image-set coefficient** | **SE** | **t** | **p** | **95% CI** |
| --- | --- | --- | --- | --- | --- | --- | --- | --- |
| root 1 | 0.876 | No | No |  |  |  |  |  |
| root 2 | 4.114 | Yes | Yes |  |  |  |  |  |
| mean minus 1sd | 2.767 | Yes | Yes | 0.195 | 0.240 | 0.813 | = .417 | [-0.279, 0.669] |
| mean | 3.927 | Yes | Yes | -0.264 | 0.169 | -1.562 | = .120 | [-0.599, 0.070] |
| mean plus 1sd | 5.087 | Yes | Yes | -0.724 | 0.240 | -3.020 | = .003 | [-1.197, -0.251] |

*Note. The lower root lies outside both the observed range and the 1 to 7 scale. In the primary unadjusted model, the estimated higher-minus-lower image-set coefficient was negative above 4.114 and its 95% confidence interval excluded zero. The region is descriptive because uniqueness threat was measured after the outcome.*

**Table S17. Gender-adjusted Study 4 Johnson-Neyman sensitivity result**

| **Point** | **Uniqueness threat** | **Observed range** | **Scale range** | **Conditional image-set coefficient** | **SE** | **t** | **p** |
| --- | --- | --- | --- | --- | --- | --- | --- |
| root 1 | 0.739 | No | No |  |  |  |  |
| root 2 | 4.172 | Yes | Yes |  |  |  |  |
| mean minus 1sd | 2.767 | Yes | Yes | 0.200 | 0.243 | 0.824 | = .411 |
| mean | 3.927 | Yes | Yes | -0.255 | 0.173 | -1.470 | = .143 |
| mean plus 1sd | 5.087 | Yes | Yes | -0.710 | 0.248 | -2.867 | = .005 |

*Note. In the gender-adjusted sensitivity model, the within-range threshold is 4.172. This close agreement with the primary threshold does not change the post-outcome measurement limitation.*

# S7. Interpretation limits that apply across the supplement

1. The archived cleaned records measure stated collaboration intention, not observed collaboration behavior.

2. Warmth and competence were recorded after the outcome, so the parallel indirect associations lack temporal precedence.

3. Study 3 threat condition is confounded with study, independent sample, and questionnaire form. The combined interaction is a cross-study comparison.

4. Study 4 uniqueness threat was recorded after the outcome. Its interaction with assigned image-set condition is correlational.

5. Study 4 robot images were not matched on familiarity, attractiveness, visual complexity, function, or eeriness in the archived record.

6. The Study 4 mapping is internally consistent with the cleaned data but is not an authenticated record of the images used during data collection.

7. The archive does not preserve the person-level reasons for 101 logged entries that are absent from the cleaned data.
